# The feasibility of the PAM intervention to support treatment-adherence in people with hypertension in primary care: a randomised clinical controlled trial

**DOI:** 10.1038/s41598-021-88170-2

**Published:** 2021-04-26

**Authors:** Aikaterini Kassavou, Venus Mirzaei, Sonia Shpendi, James Brimicombe, Jagmohan Chauhan, Debi Bhattacharya, Felix Naughton, Wendy Hardeman, Helen Eborall, Miranda Van Emmenis, Anna De Simoni, Amrit Takhar, Pankaj Gupta, Prashanth Patel, Cecilia Mascolo, Andrew Toby Prevost, Stephen Morris, Simon Griffin, Richard J. McManus, Jonathan Mant, Stephen Sutton

**Affiliations:** 1 Department of Public Health and Primary Care, The Primary Care Unit, University of Cambridge, Cambridge, UK; 2 Department of Computer Science and Technology, University of Cambridge, Cambridge, UK; 3 School of Electronics and Computer Science, University of Southampton, Southampton, UK; 4 School of Pharmacy, University of East Anglia, Norwich, UK; 5 School of Health Sciences, University of East Anglia, Norwich, UK; 6 Usher Institute, University of Edinburgh, Edinburgh, UK; 7 Institute of Population Health Sciences, Queen Mary University of London, London, UK; 8 Cambridgeshire and Peterborough Clinical Commissioning Group, Cambridge, UK; 9 Department of Metabolic Medicine and Chemical Pathology, University Hospitals of Leicester NHS Trust, Leicester, UK; 10 Nightingale-Saunders Clinical Trials and Epidemiology Unit, King’s College London, London, UK; 11 Nuffield Department of Primary Care Health Sciences, University of Oxford, Oxford, UK

**Keywords:** Psychology, Cardiology, Health care, Health occupations, Medical research

## Abstract

The PAM intervention is a behavioural intervention to support adherence to anti-hypertensive medications and therefore to lower blood pressure. This feasibility trial recruited 101 nonadherent patients (54% male, mean age 65.8 years) with hypertension and high blood pressure from nine general practices in the UK. The trial had 15.5% uptake and 7.9% attrition rate. Patients were randomly allocated to two groups: the intervention group (n = 61) received the PAM intervention as an adjunct to usual care; the control group (n = 40) received usual care only. At 3 months, biochemically validated medication adherence was improved by 20% (95% CI 3–36%) in the intervention than control, and systolic blood pressure was reduced by 9.16 mmHg (95% CI 5.69–12.64) in intervention than control. Improvements in medication adherence and reductions in blood pressure suggested potential intervention effectiveness. For a subsample of patients, improvements in medication adherence and reductions in full lipid profile (cholesterol 1.39 mmol/mol 95% CI 0.64–1.40) and in glycated haemoglobin (3.08 mmol/mol, 95% CI 0.42–5.73) favoured the intervention. A larger trial will obtain rigorous evidence about the potential clinical effectiveness and cost-effectiveness of the intervention.

*Trial registration* Trial date of first registration 28/01/2019. ISRCTN74504989. https://doi.org/10.1186/ISRCTN74504989.

## Introduction

Hypertension is a leading global modifiable risk factor for disability and premature deaths^1^. In the UK, around a third of adults have been diagnosed with hypertension^2^. Hypertension or high blood pressure is often accompanied by other health conditions, such as Type 2 Diabetes, Coronary Heart Disease and Stroke^3^. Adherence to medication can considerably reduce the health risks associated with hypertension and comorbidities; however, a substantial proportion (around 41%) of patients do not take their medication as prescribed^4^. Non-adherence reduces the effectiveness of treatment and leads to additional consultations, waste in healthcare resources, increased cardiovascular events and increased costs for the National Health Care Service^5–10^.

Patients receiving treatment for hypertension account for the majority of primary care consultations^2^. Although practitioners have an important role in advising and supporting patients’ adherence to anti-hypertensive medications^11^, their time is limited and expensive. One way to address treatment non-adherence might be for practice nurses or health care assistants to provide very brief advice and signpost patients to ongoing and low-cost support using available digital technologies, such as text messaging or downloadable applications (apps) on mobile phones^12^.

There is early evidence suggesting that text messaging interventions^13,14^ and smartphone apps^15^ can improve medication adherence in patients with long-term health conditions, but published trials are of short duration and used proxy measures of adherence. Recent trials addressed some of these limitations and found statistically significant positive effects of text messaging^16^ and smartphone apps^17^ on improving systolic blood pressure. However, there is no evidence as to whether and how treatment adherence impacted on clinical effectiveness, since medication adherence was based on self-report only.

This trial evaluated the feasibility of a behavioural intervention (the Programme on Adherence to Medication or PAM intervention) to support biochemically validated medication adherence and to therefore reduce blood pressure in patients prescribed treatment for hypertension, as an adjunct to usual care consultations in primary care. The PAM intervention is a two-component intervention, consisting of a very brief intervention facilitated by a practice nurse or health care assistant, followed by a 3-month highly tailored text messaging programme or a smartphone app^18^. The objectives of this study were to inform the design of a larger trial aiming to obtain robust evidence about the clinical effectiveness and cost effectiveness of the PAM intervention in primary care.

## Methods

### Study design

This was an individually randomised clinical controlled feasibility trial conducted in nine primary care practices in the East of England and London in the UK. All methods were implemented in accordance with the relevant guidelines and regulations. The trial protocol can be found in supplementary file 1.

### Primary care sites and patients

Nine primary care practices representing different levels of deprivation with a practice nurse who facilitated medication reviews or blood pressure checks or other relevant consultations, were recruited in this trial (for practice characteristics, see supplementary file 2 Fig. 1).

**Figure 1:**
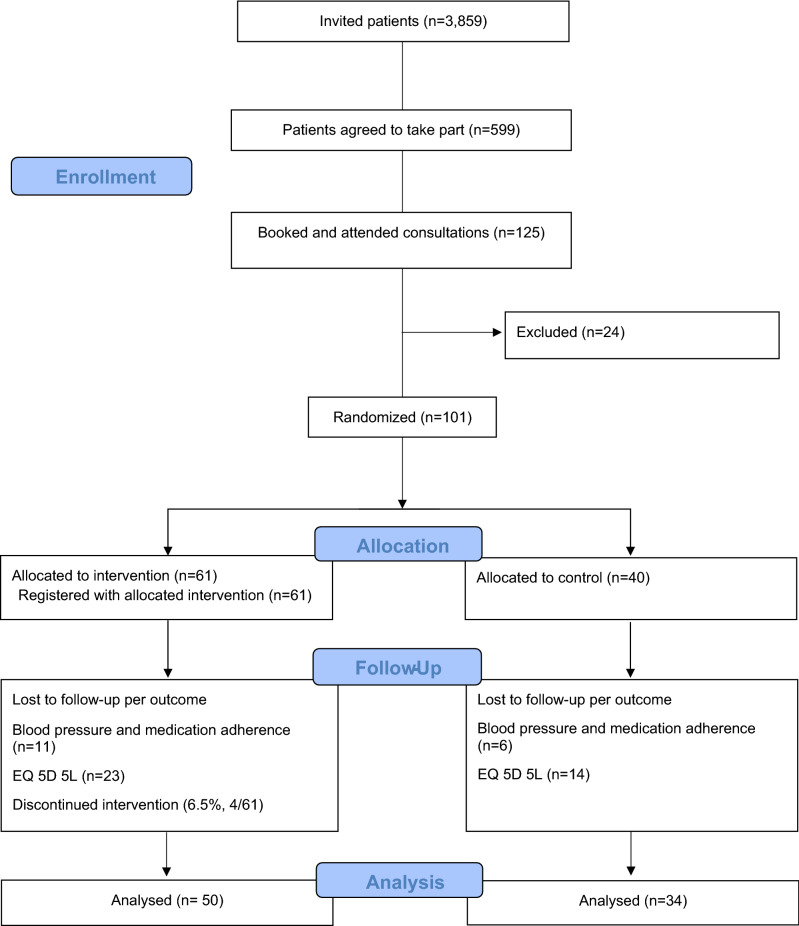
Trial flow chart.

Adults (18 years and above) receiving pharmacological treatment for hypertension in primary care, who had blood pressure above 140 mmHg/90 mmHg and were non-adherent to prescribed medication on the basis of electronic searches of practice records and practice GP assessment, were eligible for the study. Patients were not eligible if they had a long-term health condition that could impair participation or were taking part in another medication adherence or digital intervention to support treatment for long-term health conditions. Patients were also excluded based on low digital literacy (i.e., not having or using mobile phone).

Potentially eligible patients were identified using practice records and were screened for eligibility by a practice GP. Those deemed eligible were invited by post to attend the baseline consultation at their GP practice. All consultations were conducted during the morning clinics. During the practice consultation, the practice nurse accessed the Program on Adherence to Medication (PAM) online system, confirmed patient eligibility to the study, obtained written informed consent and reviewed patient medications. The nurse used the online system to then randomise participants to the intervention or the control group. Blood pressure and blood samples were collected during the practice visits.

Follow up clinical appointment was at 3 months from baseline, and it was implemented in the patient’s primary care practice by a practice nurse blinded to group allocation. Recruitment and baseline consultations were conducted from July 2019 to December 2019 and follow up consultations were conducted from October 2019 to March 2020. The study closed follow up earlier than scheduled: the trial was scheduled to complete follow up at the end of March 2020, three weeks after the practices paused consultations due to the COVID-19 pandemic.

### Randomisation and blinding

Patients were individually randomised to two groups, using a 3:2 allocation ratio (61 intervention, 40 control) using the method of random permuted blocks, with block size of 5. An unequal allocation ratio was selected to obtain more information about the intervention (e.g., intervention uptake, engagement and estimates of potential effects). Randomisation was stratified by practice nurse only. Allocation was conducted using a computer-based online randomisation tool, which generated patients’ unique ID numbers randomly. Although the allocation sequence was concealed, once a patient was allocated to a group, neither the practice nurse nor the patient was blinded to allocation.

### Intervention

The PAM intervention is a 3-month behavioural intervention consisting of one very brief intervention (VBI) facilitated by a practice nurse as an adjunct to usual care, followed by an 84-day highly tailored text messaging programme or an Android smartphone app digital intervention. Practice nurses were provided with face-to-face training on the VBI intervention by a member of the research team. One practice nurse per practice received training on the intervention. Intervention patients received the VBI and were enrolled in the ongoing digitally delivered support by registering their telephone number on the PAM online system during the practice usual care consultation. Patients were provided with a choice to receive the intervention either by a text messaging programme or a smartphone app and were given an option to switch from text messaging to the app during the intervention.

The PAM intervention was based on a theoretical framework that distinguishes between intentional non-adherence (INA) and non-intentional non-adherence (NINA)^19^, mapped onto theoretical determinants of adherence; namely, beliefs and attitudes about medication taking, self-efficacy and social norms^20^. The intervention was informed by evidence from our qualitative studies^20,21^, systematic reviews^15,22^, a previous medication adherence trial^14^, a pilot study^23^, stakeholders’ consultations and Patients and Public Involvement and Engagement^18,20^.

Following the VBI, which emphasised the importance of medication adherence, and based on information obtained from patients themselves and from practice records, patients received individually tailored messages designed to address one or both of INA and NINA reasons. Patients could provide more information and tailor the intervention messages further by responding to questions using the digital delivery modes. INA was addressed with messages to reinforce positive beliefs about taking anti-hypertensive medications (e.g., “even if you don’t feel any different after taking each of your pills, you can keep your BP under control when you take your meds regularly”), and to counter negative beliefs in a non-confrontational way (e.g., “your tablets support you to keep your blood pressure under control…”). NINA was addressed through reminders (e.g., “don’t forget to take your medication today: Ramipril, 2 tablets, 1.25 mg, at 16:00”). Other behaviour change strategies (e.g., ‘report whether or not the behaviour was performed’) are included as appropriate. A description of the intervention is given elsewhere^18^.

### Control group

Patients allocated to the control group received usual care only^12^.

### Outcomes

The purpose of this study was to establish the feasibility of a larger scale trial assessing the effectiveness and cost-effectiveness of the PAM intervention in UK primary care. This feasibility trial obtained estimates of (a) recruitment and attrition rates; (b) values for systolic and diastolic blood pressure and biochemically validated medication adherence, as well as self-reported medication adherence; (c) values for important co-morbidities i.e. full lipid profile for high cholesterol and glycated haemoglobin for type 2 diabetes; (d) quality of life and intervention implementation cost in primary care, as preliminary estimates of the intervention cost-effectiveness; and (e) intervention uptake, as well as intervention engagement and mechanisms of change.

#### Recruitment and attrition

Recruitment rate was defined as the proportion of patients responding to the trial invitations with an interest to participate, within the first two weeks from the day the invitations were sent. Attrition was measured by the proportion of patients who did not attend the follow up clinical consultations to provide complete and valid outcomes for both blood pressure and biochemically validated medication adherence at the end of the trial. Trial participation and attrition rates were recorded in primary care practice records.

#### Blood pressure

Blood pressure was measured three times using validated automated electronic sphygmomanometers. The mean of the last two blood pressure readings was used for analysis^24^. Blood pressure was measured at practice visits by practice nurse who was blinded to group allocation.

#### Biochemically validated medication adherence

Objective medication adherence was measured using biochemical testing of urine samples, which detects the presence or absence of the anti-hypertensive medications (or their metabolites where appropriate) in the urine on the screening and for a period of time up to 48 h from collection. Spot urine samples (10 ml) were collected in the morning of the clinical appointment and were transferred by a courier at room temperature from GP practices to the National Centre for Adherence Testing at the University Hospital of Leicester within 4-h (min 1hr, max 5hrs) from collection, and were stored at -70 °C until analysis. A biochemical test was undertaken by liquid chromatography-tandem mass spectrometry (LC–MS/MS)^25^. LC–MS/MS is highly sensitive and at present is the most specific technique of biochemical testing of medication adherence. Biochemical medication adherence was calculated by the number of daily doses of anti-hypertensive medications detected in the urine out of the number of daily doses of medication prescribed, accounting for adjustments at prescribed doses during the trial. Total adherence to antihypertensive medication was defined when all prescribed doses (or their metabolites where appropriate) were detected in the urinalysis, partially adherence was defined when fewer doses than prescribed were detected during analysis, and total non-adherence was defined as complete absence of any prescribed antihypertensive medication.

#### Self-reported medication adherence

Self-reported medication adherence was measured by two single items; one item measuring weekly adherence (i.e., ‘How many days in the past week have you taken all your prescribed tablets?’; scores ranging from 0 to 7, with 0 showing 0 days and 7 showing 7 days), and one item measuring monthly adherence (i.e., ‘How much of your prescribed tablets have you taken in the last month?’; scores ranging from 0 to 100%). Medication adherence was measured during practice consultations at baseline and follow up by a practice nurse blinded to group allocation.

#### Other clinical outcomes

Full lipid profile and glycated haemoglobin were measured by collecting 5 ml blood samples for each outcome, which records the cumulative values for each of full lipid profile and glycated haemoglobin during the past 3 months. Full lipid profile was measured for patients prescribed medication to treat high cholesterol, as well as hypertension; and glycated haemoglobin was measured for patients prescribed medication to treat type 2 diabetes, as well as hypertension^26^. Blood samples were trasnferred by a courier to Addenbrooke’s Cambridge University Hospital NHS Trust lab for analysis within 4 h of collection. Blood samples were collected during practice visits at baseline and at follow up.

#### Quality of life and intervention implementation cost

Quality of life was measured using the EQ-5D-5L questionnaire^27^. The EQ-5D-5L measures five Quality of Life indicators (i.e., mobility, self-care, usual activities, pain/discomfort and anxiety/depression) and total health. The questionnaire was completed by patients remotely, immediately after the baseline and follow up practice visits.

We also estimated the cost to implement the intervention in primary care. The cost was estimated accounting for the practice nurses’ training on the VBI (i.e., cost for producing the training material and the training itself) and the cost to implement the intervention as an adjunct to usual care consultations (i.e., practice nurse time to facilitate the VBI as an adjunct to usual care, primary care practice facilities utilised during the intervention, and cost for the digitally delivered support).

#### Intervention uptake, engagement and mechanisms of behaviour change

Intervention uptake and engagement were recorded automatically in the digital records. Intervention uptake was measured by the proportion of randomised patients registering for the intervention. Initial intervention engagement was measured by the proportion of randomised patients completing the VBI and the tailoring questions using the digital mode within the first 2 weeks of the intervention. Continued intervention engagement was measured by the proportion of patients completing the VBI and interacting with the digital intervention by replying to all intervention messages, excluding the tailoring questions, for a consecutive one month following the VBI. Participants who requested to stop receiving the digital intervention (i.e., those actively dropping out from the intervention) were accounted for (i.e., excluded) when calculating the score for the total continued intervention engagement.

The intervention mechanism of effect was explored using interviews and the Beliefs about Medicines Questionnaire (BMQ)^28^. Interviews are reported separately. The BMQ was completed by patients remotely (i.e., by post or online) immediately after the baseline and follow up practice visits.

### Statistical analysis

The planned sample size of 101 patients was adequate to obtain estimates for the feasibility of the intervention in primary care (e.g., 95% confidence interval widths for uptake of the intervention of at most ± 7.6% and for attrition rates of between ± 6 and ± 9% over the range 10%-30% seen in previous trials), as well as obtain values for the outcome of the main trial (i.e., systolic blood pressure) with the required precision to inform a larger effectiveness and cost-effectiveness trial. To maximise the generalisability of the intervention in primary care, this feasibility trial recruited patients from a variety of primary care practices.

The variables are summarized using descriptive statistics (Table 1). The assumptions of the analysis were met. We compared the mean systolic blood pressure, medication adherence, full lipid profile and glycated haemoglobin, at 3 months in the two arms to quantify the difference in means and 95% confidence intervals. The analysis was performed using ANCOVA on data adjusted for baseline values only. We performed the analysis to those providing complete and valid outcomes at follow up. Statistical significance tests were two-tailed, and the alpha level was 0.05. Analysis was performed during May 2020 using SPSS 26.

**Table 1:** Baseline characteristics of randomised patients.

	Intervention	Control
Age in years, mean (SD)	65 (10.6)	67.1 (11)
Gender, percent (n) male	60 (37)	45 (18)
**Index of multiple deprivation, percent (frequency)**		
10–30 (most deprived)	36.1 (22)	32.5 (13)
40–60	49.1 (30)	55 (22)
70–100 (least deprived)	14.8 (9)	12.5 (5)
**Area, percent (frequency)**		
Rural	44.3 (27)	50 (20)
Urban	55.7 (34)	50 (20)
**Blood pressure, mean (SD), mm Hg**		
Systolic	146.91 (5.33)	146.88 (6.55)
Diastolic	84.28 (7.79)	85.43 (7.42)
**Full lipid profile, mean (SD), mmol/mol**		
Cholesterol	5.37 (0.93)	5.27 (1.53)
Triglycerides	2.42 (1.25)	2.17 (0.60)
HDL cholesterol	1.93 (0.72)	1.80 (0.80)
LDL cholesterol	2.36 (0.71)	2.34 (0.75)
Glycated haemoglobin, mean (SD), mmol/mol	48.17 (9.49)	48.55 (9.88)
**Medication adherence**		
Biochemically validated, percent (frequency)	91.8 (56)	92.5 (37)
Self-reported, adherence past week, mean (SD)	5.72 (0.77)	5.78 (0.76)
Self-reported, adherence past month, mean (SD)	8.44 (1.86)	8.32 (1.52)
**Quality of life, median (min–max), EQ-5D-5L**		
Mobility	1 (1–4)	1 (1–4)
Self-care	1 (1–4)	1 (1–4)
Usual activity	1 (1–4)	1 (1–5)
Pain/discomfort	2 (1–4)	2 (1–5)
Anxiety/depression	1 (1–4)	1 (1–4)
Total health, mean (SD), EQ-5D-5L	80.25 (18.1)	71.75(23.26)
**Beliefs about medicines, means (SD)**		
Generic beliefs	23.60 (4.18)	23.58 (4.47)
Concern beliefs	16.84 (3.23)	16.81 (3.55)
Necessity beliefs	12.25 (2.96)	11.50 (3.29)
Number of defined daily doses of medication prescribed for hypertension, median (min–max)	2.00 (1–5)	2.00 (1–6)

N = 101 (n = 61 intervention, n = 40 control); blood pressure, urine samples, self-report medication adherence, Quality of Life EQ-5D-5L and Beliefs about Medicines Questionnaire. Of whom N = 42 full lipid profile (n = 28 intervention, n = 14 control) and N = 34 glycated haemoglobin (n = 23 intervention, n = 11 control). English Index of Multiple Deprivation was calculated based on practice post code. Scores range from 10 to 100. Most deprived were those located at 10–30% most deprived areas; middle deprived were those located at 40–60% least deprived areas, and least deprived were those located at 70%-100% least deprived areas. Quality of Life five dimensions score range from 1 to 5. Lower scores indicate better Quality of Life. Total health scores range from 0 to 100. Higher scores indicate better total health. Beliefs about Medicines total scores range from 5 to 25. Higher scores indicate more positive beliefs. Number of prescribed anti-hypertensives is per defined daily dose. Data are reported as percentages and frequencies, median with minimum (min) and maximum (max) values, or means with standard error (SE) and standard deviations (SD).

### Ethics approval and consent to participate

Ethical approval was sought from the North East – Tyne & Wear South Research Ethics Committee (REC reference number 19/NE/0018) and the Health Research Authority and Health and Care Research Wales. Written informed consent was signed by all participants during primary care consultations with practice nurses. All participants were informed about the aims and objectives of the study and the procedures before providing informed consent. All patients signed a consent form before their participation in this study commenced. This information included standard formulation regarding the voluntary nature of study participation as well as participants’ rights to withdraw from the study at any time without giving reason or attracting any negative consequences.

### Ethics, consent and permissions

All participants provided written informed consent to take part in this trial.

### Consent for publication

Participants’ informed consent to participate in the study included consent for publication of group-level results.

## Results

### Recruitment

In total 3859 potentially eligible patients were identified in practice records and were sent invitations to participate in the trial (Fig. 1). Of those invited, 599 patients (15.5% response rate) responded to invitations during the first two weeks from invitation, and the first 125 of them were booked and attended the baseline consultations. During baseline consultations 101 were confirmed eligible to participate and provided written informed consent. Reasons for not being eligible were patients not having or using a mobile phone regularly, a criterion that was not detected before invitation.

All patients provided complete data at baseline. The intervention and control groups were similar on all baseline variables (Table 1). Patients were on average 65.8 years of age, 54% male, with the great majority located in middle to high areas of deprivation, proportionately from urban and rural areas, and were prescribed on average 2.5 (min 1, max 6) daily doses of medication for hypertension. All patients had high blood pressure, 41% (42/101) of them had high cholesterol, 32% (34/101) of them had type 2 diabetes, and one third of them were receiving treatment for both these health conditions. Overall, patients who were non-adherent to anti-hypertensive medication based on the urinalysis tended to have higher blood pressure than those adherent to medication.

### Attrition

Three months after baseline, 83% (84/101) (50/61 intervention group, and 34/40 control group) patients provided complete and valid outcome data for both blood pressure and urinalysis for biochemical testing of adherence, giving a 16.8% (17/101) attrition rate. However, nine of these patients did not provide valid and complete outcome data at the practice for both these outcomes due to primary care practices pausing consultations during the COVID-19 pandemic, thus the attrition rate attributed to the trial is estimated to be 7.92% (8/101).

### Blood pressure

At the end of the trial, the systolic blood pressure was reduced by 9.16 mmHg (95% CI 5.69–12.64) and the diastolic by 4.85 mmHg (95% CI 1.06–8.68) in the intervention than the control (Table 2).

**Table 2:** Mean difference in blood pressure treatment.

	Baseline	Follow up	Adjusted mean difference (95% CI)
	Mean (95% CI)	Mean (95% CI)	
**Systolic**			
Intervention	146.91 (145.54–148.27)	136.90 (134.00–139.80)	9.16 (5.69–12.64)
Control	146.88 (144.79–148.98)	145.97 (144.19–147.76)	
**Diastolic**			
Intervention	84.28 (82.28–86.28)	79.55 (76.54–82.56)	4.85 (1.06–8.68)
Control	85.43 (83.06–87.81)	84.60 (81.69–87.51)	

Complete case analysis. N = 84 (n = 50 intervention, n = 34 control) for the follow up. Data present mean difference in reduction of blood pressure between intervention and control group at 3 months, adjusted for baseline values.

### Medication adherence

At the end of the trial, the biochemically validated medication adherence was improved by an average 20% (95% CI 3–36) daily prescribed doses in the intervention than control (supplementary file 2, Fig. 2). The self-reported medication adherence was improved by an average 1 day (95% CI 0.60–1.21) per week and by 1.16 (95% CI 0.56–1.74) of percent improvements by month, in intervention than control (supplementary file 2, Table 1).

**Figure 2:**
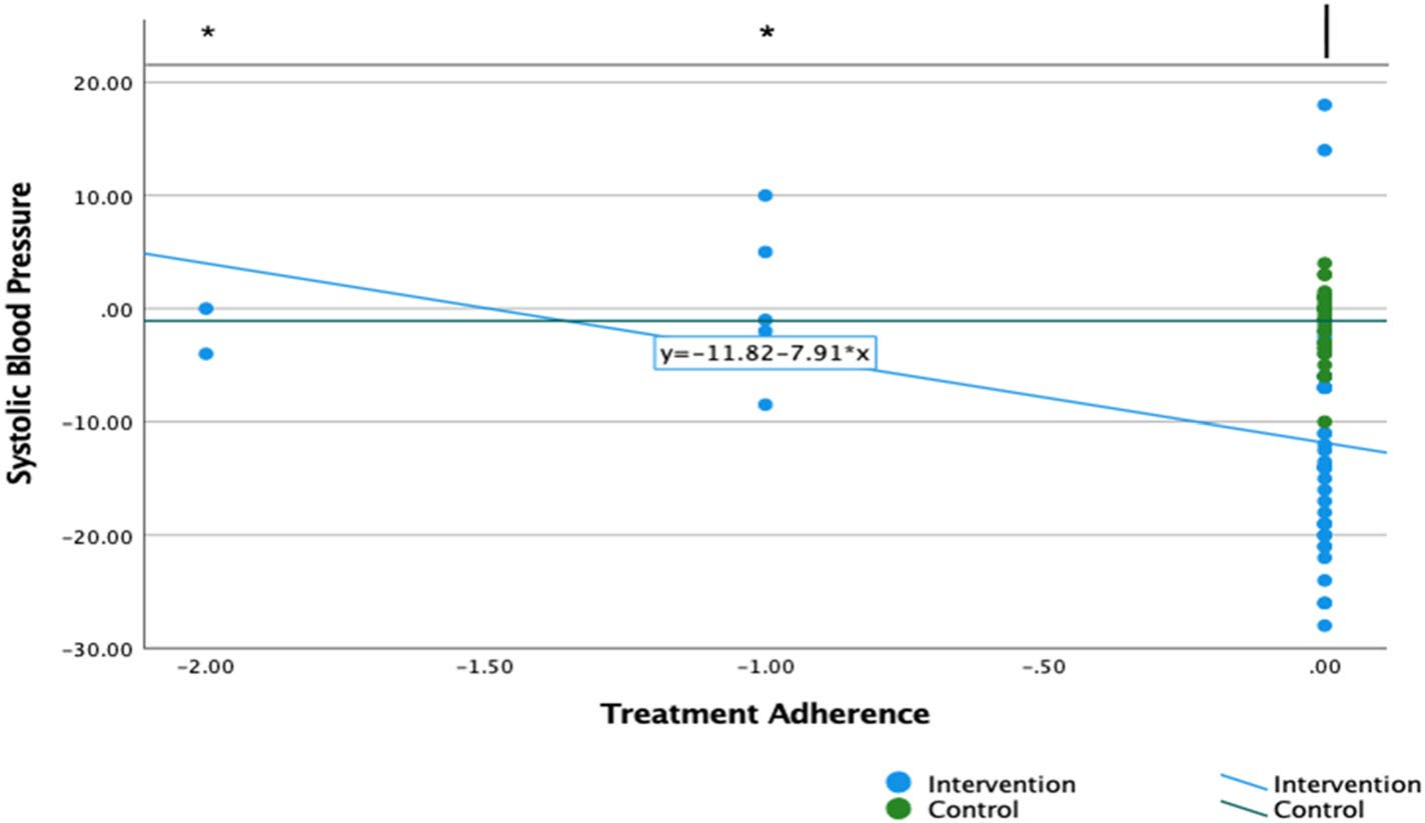
Changes in blood pressure by allocation group and biochemically validated medication adherence. Vertical axis shows the changes in systolic blood pressure. Lower numbers indicate reduction in systolic blood pressure. The horizontal axis shows the changes on biochemically validated treatment adherence. Improved total adherent is represented at level of 0.00, partially adherent at level of − 1.00 and total nonadherence at level of − 2.00. The green line is the fitted regression line for the control group, the blue line is the fitted regression line for the intervention group. The dots show the units of measure for each of the intervention or the control group. The direction of the lines shows the association between changes in systolic blood pressure and changes in treatment adherence.

### Associations between blood pressure and medication adherence

The improvements in biochemically validated medication adherence were associated with mean reductions of 10 mmHg (95% CI 7.35–13.12) in systolic blood pressure in the intervention, and with mean reductions of 1 mmHg (95% CI 0.08–2.08) in the control (Fig. 2).

Similar direction of effects were observed between the mean changes in self-reported medication adherence and mean changes in blood pressure: improvements in days of adherence were associated with mean reductions of 8.99 mmHg (95% CI 6.58–11.40) in systolic blood pressure in the intervention, and mean reductions of 2.23 mmHg (95% CI 1.07–5.54) in the control (see Supplementary file 2, Fig. 3); and improvements in monthly adherence were associated with mean reductions of 1.88 mmHg (95% CI 0.40–3.30) in systolic blood pressure in the intervention group, and mean reductions of 1.10 mmHg (95% CI − 1.26 to 3.47) in the control (see Supplementary file 2, Fig. 4).

There were no effects of the practice level variability (i.e., practice nurse facilitating the VBI or the practice IMD) on explaining the treatment effects (see Supplementary file 2, Table 2).

### Estimates of additional clinical outcomes

Intervention group patients had improved full lipid profile (cholesterol was reduced by 1.39 mmol/mol 95% CI 0.64–1.40; triglycerides were reduced by 0.66 mmol/mol, 95% CI 0.02–1.36; LDL was reduced by 0.48 mmol/mol, 95% CI 0.10–0.86) and improved glycated haemoglobin (HbA1c was reduced by 3.08 mmol/mol, 95% CI 0.42–5.73) than control (supplementary file 2 Table 1). Improvements in lipid profile and glycated haemoglobin were both associated with self-reported improvements in medication adherence, which was a more generic measure of medication adherence (supplementary file 2, Fig. 5 and Fig. 6).

### Quality of life and estimates of intervention implementation cost

There was a trend towards improvement in total health and overall quality of life for the intervention, with some improvements observed on mobility, self-care and pain/discomfort; but these were not different between groups (supplementary file 2 Table 1). The length of the VBI was on average one minute and the average intervention delivery cost was £3.5 per patient: £1.6 for the VBI and £1.9 monthly cost for the ongoing digital support.

### Intervention uptake, engagement and mechanisms of behaviour change

All patients allocated in the intervention group registered with the intervention, giving 100% intervention uptake. After the completion of the VBI, 91.8% (56/61) patients registered with the text messaging programme and 8.2% (5/61) with the smartphone app. Patients had the option to switch delivery modes and all app users switched to the text messages during the intervention. The intervention had high engagement score; 90% (55/61) of patients who completed the VBI, responded to the tailoring questions via the digital modes. With four patients actively deciding to disengage with the intervention, 72% (44/61) continued using the intervention for at least one consecutive month after the practice consultations.

At the end of the trial, beliefs about the necessity to adhere to prescribed treatment were more positive in the intervention group than the control group (b = 2.86, P = 0.004), but there were no significant differences in generic beliefs about being prescribed medications and concerns about taking medications.

## Discussion

This trial showed that the PAM intervention is feasible to support treatment adherence and adherence-related reductions in blood pressure in patients with high blood pressure in primary care. Considering that clinically meaningful improvements require sustained effect for more than 3 months, it is possible that the intervention could have clinically significant effects if its duration was longer. If these effects were sustained, they could have significant impact on preventing cardiovascular and cardiometabolic events (e.g., stroke, ischemic attack, angina).

Improvements in full lipid profile and glycated haemoglobin were found at a subsample of intervention group patients prescribed treatment for either or both high cholesterol and type 2 diabetes, in addition to hypertension. Further analysis showed a trend of the reductions in cholesterol, triglycerides and glucose haemoglobin to being associated with improved self-reported medication adherence favouring the intervention. These results suggest a positive effect of the intervention on overall treatment adherence, and thus potentially on clinical effectiveness. These findings also suggest that the intervention might be beneficial to those patients who have comorbidities, as well as to those who have hypertension only; and if these effects were sustained for longer the intervention could reduce morbidity and all-cause mortality. Improvements in other health-related behaviours that were not measured by this trial (e.g., dietary changes) could have contributed to the observed improvements in blood pressure and the additional clinical outcomes.

This feasibility trial had high uptake and low attrition rate suggesting that the PAM intervention is feasible adjunct to primary care. All intervention patients registered with the PAM intervention and the majority of them engaged with all components of the intervention for one consecutive month. The high intervention uptake suggests that PAM is a feasible adjunct to medication reviews and blood pressure checks routinely conducted in primary care. The high engagement suggests that it is likely that the positive intervention effects on blood pressure and medication adherence could be attributed to the PAM intervention.

At the end of the trial, necessity beliefs were more positive in the intervention group than in the control group, supporting the hypothesis that the intervention might has an effect at modifying necessity beliefs which in turn might impact on treatment adherence outcomes. Future research could usefully explore the potential effect of engagement with the active intervention content at modifying these beliefs, as well as intentional and nonintentional reasons of nonadherence, and thus at impacting on improved treatment outcomes. This could inform our understanding of the intervention effects and generate replicable interventions for practice.

There was no difference between groups in concern beliefs, indicating that the intervention had no impact on changing patients’ concerns about being prescribed medications. This finding highlights the challenge of improving medication adherence by addressing patients’ concerns about being prescribed pharmacological treatment.

Further analysis suggested that there were no effects of other potential confounding variables (e.g., practice level characteristics: practitioner or practice IMD) on intervention outcomes. However, a larger trial is required to detect any potential effect of practice and individual level variability on the main trial outcomes.

### Strengths and limitations

The trial found improvements in blood pressure. However, caution should be given when interpreting these results due to the lack of standardised measurement of the blood pressure across practices^24^. Future trial could usefully use a standardised method to measure blood pressure and to provide the evidence required about the effectiveness of the intervention on improving blood pressure.

Although all recruited patients were deemed non-adherent by their GP before invitation, only a small number of them were identified being non-adherent by the urine analysis at baseline. It is common for trial participants to show improved adherence results at baseline; in this trial all patients were informed about the requirement to provide urine samples for detection of anti-hypertensives before their appointment. However, the small improvement in biochemically validated treatment adherence was associated with reductions in blood pressure at follow up, suggesting that the intervention could potentially be effective.

Biochemical analysis of medication adherence is currently the most rigorous measurement of medication adherence and it detects the presence or absence of the medication, or its metabolites, in the urine for a duration of up 48 h before the collection. That said, it is possible that some patients were identified adherent based on the detection of the medication being taken once during the 48 h before the collection; taking into consideration the 4 h window for courier transport of urine samples to the lab, that is up to 40 h before the urine collection. Thus, it is possible that some patients were deemed adherent based on their daily dose of medication being taken once during the proceeding 40 h. Future analysis could usefully explore the milligrams of the medication detected in the urine or its metabolites, to usefully inform future research about medication adherence. This is particularly important for health conditions such as hypertension, where prescribed daily dose of medication should be present every 24 h to significantly impact on clinical effectiveness. A larger randomised controlled trial with adequate power to detect intervention effects on biochemical adherence could usefully explore this further and provide the evidence required about the effectiveness of the intervention.

At baseline many patients self-reported low adherence during the consultations with their health care provider. Further qualitative analysis of interviews with patients and health care providers suggested that it is likely patients to report non adherent during their consultations. The significant effects between improved self-reported medication adherence and reductions in systolic blood pressure at the end of the trial suggests that the intervention might have been effective at supporting short-term improvements in adherence and reductions in blood pressure. However, these results were obtained as part of the 3 months trial and it will require training and care to achieve the same outcomes in everyday practice.

### Future research

While the hypothesis about the effectiveness of the intervention to support improvements in biochemically validated adherence and associated reductions in blood pressure requires further research, it is encouraging that the results of this trial suggest that the PAM intervention is a feasible and potentially effective adjunct to usual care. A preliminary analysis of per-patient cost suggests that PAM is an inexpensive intervention (i.e., £1.6 for the VBI and £1.9 per month for the ongoing digital support). Considering that the intervention had short-term positive effects on clinical outcomes and no adverse effects were recorded, these results suggest that the intervention could potentially be a cost-effective solution to support treatment adherence above and beyond usual care. A future trial with adequate power will evaluate the effectiveness and cost-effectiveness of the intervention to support reductions in blood pressure and biochemically validated medication adherence as an adjunct to annual health checks to inform service provision.

## Conclusion

This feasibility trial showed that the PAM intervention is feasible and potentially effective in supporting reduction in blood pressure and medication adherence in patients with high blood pressure as an adjunct to usual care consultations. A larger trial will obtain rigorous evidence to investigate the clinical effectiveness and cost-effectiveness of the intervention.

## Supplementary Information

Supplementary File 1.

Supplementary File 2.

## Data Availability

The datasets used and/or analysed during the current study are available from the corresponding author on reasonable request.
