# Supplementary material for: The feasibility of the PAM intervention to support treatment-adherence in people with hypertension in primary care: a randomised clinical controlled trial
Source: Sci Rep. 2021 Apr 26;11:8897. doi: 10.1038/s41598-021-88170-2 (PMC8076273; doi:10.1038/s41598-021-88170-2)
Supplement: Supplementary file 1 [file 41598_2021_88170_MOESM1_ESM.pdf]

PROTOCOL

FULL/LONG TITLE

A very brief face-to-face intervention, followed by a text message and/or app intervention to support medication adherence in people prescribed treatment for hypertension in primary care. A randomised clinical controlled trial

SHORT STUDY TITLE / ACRONYM

Study specific title and acronym. i Adhere to Medication (i-AM) feasibility trial of the PAM intervention

PROTOCOL VERSION NUMBER AND DATE

Version: 03

Date: 19.10.18

**RESEARCH REFERENCE NUMBERS**	
**IRAS Number**	252054
**FUNDERS Number:**	PB-PG-0215-36032
**REC reference:**	19/NE/0018

This protocol adheres to the HRA and Medical Research Council's guidance and order of content.

**KEY CONTACTS**	
**Chief Investigator**	Professor Stephen Sutton<br>University of Cambridge, School of Clinical Medicine, Institute of Public Health, Forvie Site, Cambridge, CB2 0SR<br>Phone: +44 1223 330594<br>Email: srs34@medschl.cam.ac.uk
**Co-investigators**	Co-investigators listed in the grant application.<br>Prof. Jonathan Mant, Prof Simon Griffin, Dr Wendy Hardeman, Dr Felix Naughton, Dr Anna De Simoni, Dr Helen Eborall, Prof Andrew Toby Prevost, Prof Stephen Morris, Dr Debi Bhattacharya, Prof. Celiclia Moscolo, Dr Amrit Takhar, Prof Richard McManus.
**Protocol design and contact details**	Dr Katerina Kassavou<br>University of Cambridge, School of Clinical Medicine, Institute of Public Health, Forvie Site, Cambridge, CB2 0SR<br>Phone: +44 1223 330456<br>kk532@medschl.cam.ac.uk
**Sponsors**	University of Cambridge<br>Cambridgeshire and Peterborough CCG
**Funder**	NIHR, Programme Grants for Applied Research

SUMMARY		
Abstract	This research aims to assess the feasibility of a very brief face to face consultation (VBI) followed by a text message or app intervention aiming to support adherence in patients prescribed antihypertensive medications in primary care. The study aims to generate evidence to decide whether and how to proceed with a full-scale effectiveness and cost effectiveness trial. Thus, the focus of this trial is on assessing implementation procedures, including fidelity, as well as on obtaining estimates of effectiveness and cost-effectiveness. This means, that we will estimate recruitment, uptake and attrition rates, as well as potential effectiveness and cost effectiveness of the intervention at improving blood pressure and medication adherence. The intervention is based on theory and evidence, including results from a randomised controlled trial (MAPS intervention). We will aim to refine parameters of our MAPS intervention and adapt intervention content to a new delivery mode (i.e., app). If successful, this intervention will provide highly tailored support to large numbers of patients who could experience short- and long-term health benefits by taking their medications as prescribed. Given its large reach, the proposed intervention could have a substantial public health impact. The NHS would also benefit from the low cost of implementing the intervention and patients' satisfaction with the health care provided.	
Participants	Practitioners (practice nurses or health care assistants)<br>Patients with diagnosis of hypertension and high blood pressure. Patients with comorbidities of hypertension, type 2 diabetes and cholesterol, will also be eligible for inclusion.	
Sample size	N=109 (n=100 patients and n=9 practitioners)	
Duration	1 year and 3 months	
Aims and objectives		Main outcome Measures/ Sources of data collection
Assess the feasibility and obtain estimates of effectiveness and cost-effectiveness of the intervention in a randomised clinical controlled trial		Practice database records<br>Research team records<br>Log-files (text message and app)<br>Self-reported questionnaires<br>Blood pressure (and blood samples for a subsample of patients)<br>Urinalysis<br>Cumulative Medication Gap<br>Follow up Interviews

LIST of CONTENTS

**GENERAL INFORMATION**	**Page No.**
TITLE PAGE	1
RESEARCH REFERENCE NUMBERS	1
KEY CONTACTS	2
SUMMARY	3
LIST of CONTENTS	4
LIST OF ABBREVIATIONS	5
FLOW CHART	6
**SECTION**	
BACKGROUND AND RATIONALE	7
AIMS AND OBJECTIVES	9
SAMPLE	10
RECRUITMENT	11
STUDY SETTING AND STUDY IMPLEMENTATION PROCEDURES	13
STUDY BASELINE AND FOLLOW UP MEASUREMENTS	14
STUDY OUTCOMES AND STUDY MEASUREMENTS	15
DATA ANALYSIS	18
RESEARCH MANAGEMENT AND GOVERNANCE ARRANGEMENTS	18
ETHICAL AND REGULATORY CONSIDERATIONS	19
PATIENT AND PUBLIC INVOLVEMENT	23
DISSEMINATION STRATEGY	24
REFERENCES	24

LIST OF ABBREVIATIONS AND TERMINOLOGY	
CRN	Clinical Research Network
HBP	High Blood Pressure
DT2	Diabetes Type 2
NICE	National Institute of Health and Care Excellence
Participants	Practitioners and patients
Practitioner	Practice nurse or health care assistant
Patients	People prescribed medication for HBP, or comorbidities of HBP, type 2 diabetes or cholesterol
Consultations	Consultations conducted by practitioners to patients
VBI	Very Brief Intervention, refers to very brief behavioural interventions
SMS	Short Messaging Service
APP	Application
MAPS	Medication Adherence for Patients Support
BCTs	Behaviour Change Techniques
IMD	Index of Multiple Deprivation
SIP	Study Information Pack
SystmOne	Practice database and practice computers system (terms are used interchangeably)
Emis	Practice database and practice computers system (terms are used interchangeably)
EQ-5D	Health Related Quality of Life by the EuroQol
BP	Blood Pressure
CMG	Cumulative Medication Gap
HbA1c	Glycated Haemoglobin
NINA	Non Intentional Non Adherence
INA	Intentional Non Adherence
MARS	Medication Adherence Rating Scale
LC-MS/MS	Liquid chromatography-tandem mass spectrometry
PDCT	practice data collection tool or practice database records refers to log file completed by practice staff about study data collection
RTR	Research team records refers to log file completed by the research team about study data collection
PPI	Patient and Public Involvement
NIHR	National Institute for Health Research

STUDY FLOW CHART

Eligible practices are approached by the CRN; 9 recruited

↓

Eligible practitioners (n=9) complete the training about the face-to-face consultation

↓

Eligible patients receive information about the study and are invited to the face-to-face consultation

↓

Interested patients attend face to face consultation,

provide informed consent, and baseline data

↓

Consented patients (n = 100) are randomised to intervention or comparator group

and receive information about group allocation

↓

Intervention group (n=60)
Receive very brief intervention (VBI) by practitioner, information about how to use text message and/or app

↓

Receive highly tailored and interactive text messages and/or app intervention for three months

↓

Comparator group (n=40)

Receive information about usual care

↓

Follow up at three months (from informed consent).

All patients attend the primary care practice and provide follow up data;

and complete self-reported questionnaires.

A subsample of patients (n=25; 15 intervention and 10 comparator group, at three months post randomisation) and all practitioners (n=9, at the completion of the face-to-face consultations) will be invited at a follow up interview.

STUDY PROTOCOL

Feasibility of a very brief face to face consultation followed by a text message or app intervention to support adherence to anti-hypertensive medications in primary care. a randomised clinical controlled trial

BACKGROUND AND RATIONALE

In England, there are 13.5 million adults diagnosed with Hypertension and more than 5.5 million are undiagnosed [1]. Hypertension (High Blood Pressure; HBP) accounts for 75.000 deaths, and it is often accompanied with diabetes type 2 (DT2), cholesterol, and other health conditions. Hypertension is a major risk factors for disability and premature death, and medication adherence can considerably lower these risks [2-3]. However, substantial proportions of patients do not take their medication as prescribed [4]. Non-adherence reduces the effectiveness of treatment, wastes healthcare resources and leads to additional consultations, referrals, investigations, prescriptions and hospital admissions. In England, non-adherence to HBP treatment is estimated to cost the health service over £390 million per year [3]. A Cochrane review of interventions for medication adherence concluded that current interventions are not very effective and called for better interventions evaluated using rigorous trials [5]. Both a NICE guideline [6] and a report commissioned by the Department of Health [3] have recommended that novel and cost-effective interventions for medication adherence should be developed and tested.

Setting. The clinical management of hypertension and comorbidities is one of the most common interventions in primary care; and pharmacological treatment is essential part of effective hypertension management [7]. Primary care practitioners have an important role in advising, encouraging and supporting patients to adhere to their prescribed treatment. Practitioners complete consultation with HBP patients at least one per year, or even more often. The recent update from Public Health England suggests that practices embed blood pressure monitoring into routine care [1]. Moreover, patients with comorbidities, such as HBP and heart failure, are invited to attend practitioners' consultations every three months. Patients are invited to attend consultations, when a new medication is prescribed, or dose of medication is adjusted. Considering that primary care practitioners are the first point of contact for patients with HBP or comorbidities, NICE recommends practitioners to deliver brief or very brief behaviour change interventions (VBIs) as part of usual care and signpost patients to options for ongoing and low-cost support [8,9].

One way to address the challenge of non-adherence and provide ongoing support to patients is to provide patients with a VBI, followed by digital interventions such as text messaging (sms) or downloadable applications (apps) on mobile phones. Text messaging and apps are popular modes of communication in the UK. During 2016, more than four in five adults (83%) used traditional mobile messaging services [10], and the number of downloaded apps is expected to reach 20 billion by 2020 [11]. The wide use of such technology highlights the potential of the intervention to reach large numbers of patients, as an adjunct to primary care consultations.

Very brief face to face intervention (VBI). For this study, very brief intervention (VBI) refers to a time-constricted (>5min), patient-centred intervention which aims to change health behaviours. Interventions delivered by primary care nurses are effective at supporting decreases in patients' blood pressure [12,13] and are of low cost [14], especially if delivered using very brief advice. Previous trials suggest that nurse can effectively facilitate behavioural interventions to improve adherence to anti-hypertensive medications [15]. One behavioural intervention delivered face to face by a nurse in a single 30-minute session, found to effectively increase medication adherence, with no adverse effect and treatment satisfaction, compared to usual care [16]. Although nurse-led interventions may be a promising way forward, no trials have examined their efficacy to deliver VBI to support adherence to anti-hypertensive medication and reductions in blood pressure.

Digital interventions: text messaging and apps. A meta- analysis of 16 randomised clinical trials with 2,742 patients showed that text messaging significantly improved medication adherence compared with controls [17]. In addition, a 2016 Cochrane review identified seven trials comparing a text message intervention with controls for chronic medication adherence, with every study reviewed finding text message interventions to improve adherence, and the review finding text messages to approximately double the odds for adherence to medication [18]. Results from our recent systematic review of digital interventions, including text messaging trials targeting adherence to anti-hypertensive, suggest that text message interventions can double the odds of adherence (OR=1.9, 95%CI 1.5-2.3) when compared to usual care [19]. However, the review highlighted the lack of long-term interventions with rigorous outcome measures. To our knowledge, there is only one rigorous text messaging trial, which has proved to be effective at supporting adherence to anti-hypertensive medications, but it was conducted at a different health care setting [20]. However, evidence form our recent medication adherence feasibility trial with 140 patients (called MAPS) suggested that a text message intervention is feasible in the UK primary care setting [21].

Results from our recent review on medication adherence trials, delivered by apps, suggested that such interventions can significantly increase adherence to prescribed medications (OR 2.120, 95% CI 1.635-2.747, k=9), compared to usual care or minimal app-based intervention. However, there were no evidence on the optimal content and delivery of these interventions, and the effects were limited to short-term and self-reported outcome measurements [22].

DESCRIPTION OF THE INTERVENTION

The proposed intervention is based on the theoretical framework that distinguishes between intentional non-adherence (INA) and non-intentional non-adherence (NINA) [23], mapped onto theoretical determinants [24,25]. The intervention is informed by our qualitative findings [26], systematic reviews [19, 22], as well as findings from our current MAPS feasibility trial [21]. The proposed intervention has adapted the parameters from MAPS underpinning theory and Behaviour Change Techniques [27], and adjusted content and messages to a new delivery mode (i.e., VBI followed by a text message or app, instead of text and voice messages).

Based on information obtained from patients themselves and practice databases, patients will receive individually tailored advise and messages designed to address one or both of INA and NINA reasons. INA will be addressed with messages to reinforce positive beliefs about medications (e.g., "my high blood pressure tablets help to control my blood pressure levels"), and to counter negative beliefs in a non-confrontational way (e.g., "Some people believe that it's not necessary to take these tablets every day. But it is important not to miss a dose because..."). NINA will be addressed through (a) explicit (e.g., "It's time to take your evening tablet") or implicit reminders; (b) action plans/implementation intentions where participants record where, when and how they will take their tablets as prescribed, especially when participants are anticipating a change in routine. Other behaviour change techniques (BCTs) will be included as appropriate (e.g., 'report whether or not the behaviour was performed', 'information on social and environmental consequences').

STUDY AIMS AND OBJECTIVES

This study will build upon the positive findings of the literature and our MAPS intervention and assess the feasibility of a VBI followed by text messaging or an app intervention to support adherence to anti-hypertensive medications and thus to improve blood pressure in primary care. The study aims to generate evidence to decide whether and how to proceed with a full-scale effectiveness and cost effectiveness trial. This means that the study will estimate recruitment, uptake and attrition rates, as well as obtain preliminary estimates of intervention effectiveness and cost effectiveness in primary care.

Aim: this study aims to assess the feasibility of PAM intervention in primary care.

Objectives, are to estimate:

a) Recruitment and attrition rate
b) Uptake and retention
c) Estimates of intervention effectiveness and cost effectiveness
d) Implementation procedures, including fidelity and engagement

SAMPLE

Primary care practices. Nine practices will be recruited (Five in East of England, four in East London). Practices will be included if they have at least 400 eligible patients, at least one eligible nurse or healthcare assistant (for nurses and health care assistants' eligibility, see below) and use SystmOne or EMIS practice computer system. We will aim to recruit practices with a range of deprivation levels (defined by the Index of Multiple Deprivation derived from the practice post code; at least one in high and one in low IMD). To maximize scalability, we have not specified additional eligibility criteria.

Practitioners: practice nurses or health care assistants.

Practitioners will be included if they advise patients with hypertension during annual reviews, medication reviews, blood pressure checks or similar consultations. We will recruit at least one practitioner per practice.

Patients with high blood pressure. We will recruit in total 100 patients: on average 17 patients per practice, n=10 for the intervention and n=7 for the comparator group.

Patients will be included if they: (a) have a diagnosis of HBP, or comorbidities of HBP type 2 diabetes cholesterol; (b) are prescribed at least one antihypertensive medication (e.g., ACE inhibitors, beta blockers, calcium channel blockers, diuretics, alpha1 blockers, alpha2 agonists) for at least six months before study recruitment, as confirmed by practice records; (c) have poorly controlled HBP, as indicated by clinical measures (i.e., for treated HBP: blood pressure >140/90 mmHg if under 80 years old and >150/90 mmHg if over 80 years old. For people self-monitoring HBP at home or have white coat effect >135/85 mmHg if under 80 years and >145/85mmHg if over 80 years) during the last six months; or gaps in ordering or filling repeat prescriptions (i.e., Cumulative Medication Gap: CMG >0.20 for a duration of three months before recruitment); (d) have a good understanding of English; (e) are able to use mobile phones; (f) have the capacity to provide informed consent.

Patients will be excluded if they: (a) have blood pressure > 200/100mm Hg, postural HBP (>20mm Hg systolic drop), (b) have a diagnosis of dementia, aphasia or other cognitive difficulties that could affect study participation; (c) have had a recent severe life-threatening event or are under treatment for another long-term health condition (e.g. cancer); (d) receive kidney dialysis; (e) take part in another study; (f) plan to move from the area in the next 6 months, (g) BP not managed by their GP practice.

RECRUITMENT

Practitioners. The practice manager will provide eligible practitioners with the invitation letter and information sheet. Interested practitioners will contact the research team and provide electronic consent to participate. Eligible practitioners will receive a training to the face-to-face consultation, which includes the VBI, a study online system and information about study implementation procedures. Practitioners will complete the training online (2 hours) and face-to-face with a member of the research team (1-hour). Practitioners will be allocated study ids by the research team. Informed consent for practitioners will also include their consent to be interviewed at completion of recruitment and the face-to-face consultations with the patients.

Patients. Patient's recruitment strategies will be proactive and opportunistic.
During proactive recruitment, all eligible patients will be identified through searches at the practice records by the practice manager and checked for eligibility by the practice GP. A note for study eligibility will be included in the practice computers systems for all eligible patients.
Proactive recruitment methods will include face-to-face, postage invitations, text messages or emails and opportunistic recruitment will include face-to-face, posters, advertisements (at newsletters and patients' repeat prescription) and social media videos (at practice electronic monitors, websites, electronic repeat prescription sites, e-newsletter, practice twitter or facebook accounts).
Face-to-face recruitment. The practitioner will approach eligible patients face-to-face during annual reviews, medication reviews, blood pressure checks or other similar usual care consultations. The practitioner will be reminded to recruit patients using the reminder feature of the practice computer system (e.g., SystmOne or EMIS: a reminder on the computer screen will appear along with the details of the eligible patient, during the consultation). The reminder feature will include information about participant's eligibility to the study (e.g., high blood pressure hypertension registry). Patients who have not been identified during practice records eligibility checks, but they are deemed eligible to participate by the practitioner, they will be recruited opportunistically. During the opportunistic face-

to-face consultations, the practitioner will provide patient with the Study Information Pack (SIP), which includes all information needed for the patient to make an informed decision about study participation. This information will be provided in written (e.g., in patient information sheet) and/or using videos in electronic devices (e.g., iPads, kindles).

During the face-to-face recruitment, and if the patient is interested to proceed, the practitioner will open the PAM study online system and confirm eligibility criteria. If the patient is confirmed to be eligible, the system will generate a unique id number. The study online system will then prompt the practitioner to ask the patient for any questions, and when these are answered, the practitioner will obtain written consent form.

If the patient would like more time to think about the study, the practitioner will book another appointment with the patient or will obtain patient's verbal consent to pass contact details to the research team. If patient prefers to be contacted by the research team, a member of the research team will call patients (at least 24 hours later), ask for their decision on the study and arrange the collection of the consent form.

<u>Postage, text messages, emails</u>. The practice will use postage, text or email (based on practices available resources) to invite a subsample of eligible patients for an appointment with the practitioner. The Study Information Pack (SIP) will include the invitation letter, the information sheet, and video explaining the study procedures (i.e., study procedures video).

For the postage recruitment, the invitation letter and SIP will be provided in written format (the invitation letter will also include a url link and a QR code to access at a Qualtrics website that will include the invitation letter, the information sheet, the study procedures video, and a form to express interest to participate). For the text message and emails, the SIP will be provided in electronic format (patients will be sent a link to a Qualtrics website that will include the invitation letter, the information sheet, the study procedures video, and a form to express interest to participate). Patients who express interest to participate they will be contacted to arrange an appointment with the health care practitioner, where written informed consent will be obtained.

If eligible patients have not responded to the invitation within two weeks, then a member of the practice staff will call eligible patients to ask whether they would like to take part in the study. In this case, patients will be asked to book an appointment with the practice. If patients require more time to think about their participation in the study, the practice staff will obtain their verbal consent to pass their contact details to the research team.

If patients have already booked an appointment with their GP practice (e.g., for a medication review or annual review), the practice will send the SIP before that meeting. Similar to the process reported above, during the consultation with the interested patient, the practitioner will confirm eligibility criteria and obtain informed consent.

The opportunistic recruitment (apart from the face-to-face described above) it will include a social media video, printed posters, flyers and adverts. This opportunistic recruitment material will include patients' eligibility criteria and invitation to the study. The social media video will be displayed at the practice and dispensary waiting areas, and at practice website, twitter, facebook and e-newsletters (based on practices available resources). The printed poster and the flyer (smaller version of the poster) will be displayed at the practice and pharmacy dispensary waiting areas. It will also include links and QR codes to study procedures video. For practices with self-monitoring BP devices, printed recruitment material (e.g., poster, flyers) will be placed at those areas. Recruitment material will prompt patients to contact their practice nurse, if they are interested to participate. Contact details of the research team will also be provided. If the patient contacts the research team, the research team will respond to any question patient has and facilitate patient to book an appointment with their practitioner at the GP practice.

STUDY SETTING AND STUDY IMPLEMENTATION PROCEDURES

Practitioners will receive a training package on how to facilitate the face-to-face consultation, which includes: the introduction to the study, obtaining informed consent, using the PAM online study system, randomize patients to intervention or comparator group, facilitate the VBI to the intervention group patients and signpost them to the digital intervention, and facilitate information about follow up study procedures to all patients.

The practitioners will be sent the training online and asked to work through the training package on their own, before the meeting with the research team. During the face-to-face meeting with a member of the research team, the practitioner will be asked to work through the training package with the researcher using role play. Practitioners will be asked to practice at least once on their own after the meeting with the researcher and before they initiate the real time consultations with the patients.

During the face-to-face consultation with the patients, and after informed consent, practitioner will obtain baseline data and will inform patient about group allocation. At that point both the practitioner and the patient will become aware of patient's allocation group. Contamination between groups will be assessed by allocating patients from the same address in the same group. Patients will be asked

about whether they know anyone else who is participating and which group they are in. After group allocation, the practitioner will provide intervention group patients with the VBI and will signpost them to the digital intervention; an option of text message or app and will provide them with information on how to use the digital intervention. The study online system will facilitate intervention delivery (i.e., VBI and signposting). The information about the digital intervention will be provided written and video format (i.e., intervention video, a video on how to use the text message or app). Intervention group patients will receive the text message or app for a duration of three months. Patients who will select to receive the intervention using text messages, they will be provided with the option to switch to the app during the three months intervention. Comparator group patients will receive usual care only. All patients will receive information about study procedures at follow up in written and video format.

STUDY BASELINE AND FOLLOW UP MEASUREMENTS

Baseline. At baseline and before randomisation, patients will provide blood pressure (BP) readings (if they have hypertension only) or BP and blood samples (if they have hypertension plus type 2 diabetes and/or cholesterol). BP and blood samples (one sample if they have type 2 diabetes only, or two sample if they have type 2 diabetes and cholesterol) will be collected by the practitioner during the practice appointment. Practices will send anonymized results electronically to the research team.

Patients will also be asked to provide urine samples during baseline practice visits; they will be provided with a urine sample kit and explained how to collect a sample of approx. 10ml and return this to the practice. Practice staff will post the urine samples to Leicester lab for analysis for measuring usage of anti-hypertensive medication [28]. Self-reported medication adherence will be assessed before randomisation using the Medication Adherence Rating Scale (MARS) [29] and two single items during the face-to-face consultation.

Patients will also be asked to complete a brief questionnaire, including the EQ-5D-5L [30] and Beliefs about Medicine Questionnaire (BMQ) [31]. Patients will be provided with the option to complete the brief questionnaire and post this to the research team (using reply paid envelopes) or complete it online using Qualtrics (sent to patients' email after informed consent).

Follow up. Three months after baseline, a practitioner will contact the participant for a follow up consultation, where the practitioner (i.e., not the practitioner who facilitated the baseline consultation) will collect clinical outcome measures, similar to those collected at baseline (BP; blood

sample for full lipid profile and HbA1c) and medication adherence outcomes (urine sample, self-reported medication adherence). The research team will send patients (using the post or electronically) self-reported questionnaires; e.g., EQ5D-5L, MARS, BMQ, and items measuring the utility and satisfaction with the digital intervention (for the intervention group only). Practice staff will extract data on usage of primary care resources and patients' refill prescriptions from practice records.

Post-trial interviews with patients: A subsample of intervention (n=15) and control (n=10) group patients will be invited to attend a follow up face-to-face or telephone semi-structured interview with a member of the research team. For pragmatic reasons, we will invite the first 15 intervention and first 10 control participants who complete the follow up. The rationale for including interviews with the control group is that, in a similar way to quantitative mediation analysis, it is helpful to include people who have not received the intervention as well as those who have.

The aim of the interviews with the intervention group is to explore patients' experience with the intervention and gain recommendations for improvement. Specifically, interviews will aim to explore views on intervention content and delivery mode and obtain recommendations about the potential mechanism of action. The semi-structured design will allow the identification of participants' thoughts about the barriers and facilitators to intervention content and delivery mode. All patients will also be asked about usage of practice resources during the three months intervention. The researcher will use prompts to facilitate responses.

Post-trial interviews with practitioners. Practitioners who complete PAM consultations will be invited to a semi-structured follow up interview to obtain their views and recommendations about the PAM consultation as an adjunct to usual care, the VBI and signposting, and use of practice resources related to the study.

STUDY OUTCOMES AND STUDY MEASUREMENTS

The study will evaluate trial implementation procedures, intervention impact and cost, all of which will inform the decision on whether to conduct a larger cost effectiveness trial. Specifically, we will evaluate the following outcomes:

Sociodemographic measures. Participants age, gender, ethnicity, marital status, employment, education and postcode will be assessed at baseline only.

Recruitment rate. For recruitment rate, we will monitor: (a) number of practice nurses and of eligible patients invited to the study (recorded by practice staff in practice data collection tool; PDCT); (b) number of nurses' views of online training and number of patients' view of the study intervention videos (recorded by frequency and duration of videos viewing in research team records; RTR); (c) proportion and demographics of nurses who contact the research team and of patients who contact the practice and/or the research team to express interest to participate (recorded by practice in PDCT or research staff in RTR); (d) proportion and demographics of nurses (recorded by research team in RTR) and patients (recorded by the practitioner in SDCT) who provide informed consent. Patients' recruitment rate of >= 15% is set as threshold to indicate study feasibility to recruit.

Study uptake. For practitioners' uptake, we will monitor (in RTR) the number and demographics of nurses who complete the training and those who recruit at least half of the requested number of patient (n≥9). For intervention group uptake, we will monitor the number and demographics of intervention group patients who provide complete baseline data, complete the VBI (recorded by study online system), and respond to at least one intervention message (for text messages) or download the app (for app) during the first week of the digital intervention (data automatically recorded in digital log files). For comparator group uptake, we will monitor the number and demographics of comparator group patients who provide complete baseline data and complete the face-to-face consultation (recorded by study online system). Uptake rate of >= 80% for practitioners and >=70% for patients is set as threshold to indicate intervention feasibility to recruit.

Fidelity and engagement. We will audio-record a subsample of 25 randomly selected practitioners' consultations to assess fidelity of the consultations. Practitioners will be notified about the audio-recording by the study online system, after they have completed at least six consultations.
We will record: (a) the frequency and duration of practitioners' viewings of the online training package before patient consultation, (b) the frequency of text messages and/or app usage (i.e., responses to query messages), and (c) patients' usage of app features (for app only). We will also monitor the number of text messages or app notifications sent and the number and content received by patients (automatically recorded in digital log files).

We will explore views and recommendations about study implementation procedures and intervention impact on medication adherence and health care for hypertension (during follow up interviews with nurses and patients). Specifically, we will aim to obtain nurses' views and recommendations about the training material and the baseline and follow up study implementation

procedures. We will also obtain patients' views and recommendations about the specific elements of the intervention (e.g., content and duration of the VBI; content, frequency and duration of the text messages or app notifications), and whether and how these might have impacted on medication adherence and clinical outcomes.

Attrition rate. We will record the proportion and demographics of patients who (a) provide valid outcome measures at three months follow up, and (b) register to intervention group but then decline further messages (intervention dropout). Attrition rate of >=80% at three months follow up is set as a threshold to indicate study feasibility to collect outcomes at follow up.

Medication adherence. Urine sample analysis will be conducted by HP LC-MS/MS (liquid chromatography-tandem mass spectrometry) to objectively determine usage of anti-hypertensive medication (baseline and follow up).
Self-reported medication adherence will be measures by MARS and two single items (baseline and follow up). Refill medication will be assessed using the Medication Cumulative Gap (three months before randomization, during intervention and at follow up).

Blood pressure and other clinical outcomes. Blood pressure will be measured using standard validated automated electronic sphygmomanometer (i.e., Omron) blood pressure monitors, similar to the TASMINHS2 trial [32] and MRC guidelines [33]. Full lipid profile and glycated haemoglobin (HbA1c) will be measured by blood samples.

Health economic evaluation/cost. We will record health resources use (e.g., additional practice visits, medication, and referrals) and research resource use (e.g., frequency and duration of contact for queries relevant to the study) from practice records and from practitioners and patients follow up interviews. We will measure social care resource use using a patient cost questionnaire and from practitioners and patients follow up interviews. We will also measure health utility using the EQ-5D-5L, intervention utility and satisfaction (self-reported questionnaires, intervention group only), and medications and refill medications cost.

DATA ANALYSIS

To analyse practitioners' consultations, we will develop a coding frame and we will code the consultation content. The coding frame will be mapped on the objectives and techniques designed to facilitate the consultation and the VBI. We will also code thematically any additional content delivered during the consultations and explore whether and how it might have influenced implementation procedures and intervention impact (e.g., contamination).

A study statistician will write a detailed statistical analysis plan for the quantitative data and will conduct the analyses, blinded to group allocation, at the completion of the study. The main quantitative data will be expressed as proportions with 95% confidence intervals or means with standard deviations. We will use chi-squared tests and ANCOVA to examine differences between allocated groups. Analysis will be on intention to treat basis for complete cases. A sensitivity analysis will examine potential effect of missing data (e.g., missing data will be computed by the most recent previous data or by mean of the series). The differences between the intervention and comparator group in adherence and clinical outcomes will be calculated using data adjusted for baseline values. Systolic blood pressure and adherence to anti-hypertensive at follow up will be the main outcomes: analysis of these outcomes will compare changes between the intervention and comparator group adjusted for baseline BP or medication adherence. We will also explore potential impact of practice level variables (e.g., practitioner, practice IMD). Descriptive statistics will explore changed Quality of adjusted life years (QALYs). Subgroup analyses will explore changes at theoretical determinants (e.g., INA, NINA, self-efficacy) between the intervention and comparator group. Statistical significance will be set at $p < 0.05$. Multi-perspective thematic analysis will be used to analyse the semi-structured follow-up interviews with practitioners and patients [34,35].

RESEARCH MANAGEMENT AND GOVERNANCE ARRANGEMENTS

The management of the study will be undertaken by the research team based in the Primary Care Unit at the Institute of Public Health at the University of Cambridge. Katerina Kassavou will lead the programme, will be responsible for overall programme management and will supervise the researchers who will assist with the day-to-day running of the research. Study progress will be closely monitored by the research team who will hold weekly study review meetings and instigate any amendments required. The research team has no conflict of interest.

Sponsor: The University of Cambridge will act as the sponsor for this study. The Cambridgeshire and Peterborough Clinical Commissioning Group (CCG) is a co-sponsor.

ETHICAL AND REGULATORY CONSIDERATIONS

Benefits to the participants and the NHS

The face-to-face intervention delivered by the practitioners has been designed to facilitate patients' reasons of medication non-adherence and to enrol them into the text messaging or app intervention. We will use this information to make recommendations to health care providers in primary care about how best to support people take their prescribed medications by delivering a very brief advice.

The text messaging service and app intervention has been designed to reflect research which suggest digital interventions may enhance medication adherence and reduce blood pressure, by providing ongoing support following practitioners' consultations. This intervention will be available to provide advice on taking medications and generic health care. It is anticipated that this element of the digital intervention will increase patients' feelings of satisfaction with the continuous care they receive from the primary care practice. If feasible, this low-cost intervention could reach people even in the most deprived areas. Practitioners may not directly benefit from the study, but if successful, this intervention may benefit people with long-term health conditions and help other practitioners to achieve practice targets.

Participants in this study will contribute to the design of a low-cost intervention. This might not directly benefit them, but if feasible, this intervention will have the potential to reach large number of people and provide them with highly tailored support.

Furthermore, medication non-adherence reduces the effectiveness of treatment and increases the cost to the NHS from hospital admissions, additional consultations, referrals, investigations and medicine wastage. Thus, if feasible we will further test the efficacy of this intervention. If this scalable intervention is effective, it will most probably will be a cost-effective intervention for the NHS.

All participants will benefit by the results and recommendations made from participants of this research, which will be available to them upon completion of the study.

Potential risks to the participants and how to minimise them.

This research project does not involve the test of any medical device/equipment or drugs. Thus, in our view, this is a low-risk study. The feasibility study evaluates a psychological/behavioural intervention delivered for three months. The intervention involves messages to provide advice and support on medication taking behaviour. The main ethical issues apply to the process of recruitment, gaining informed consent from participants, the confidentiality of the data provided, the anonymity of the research and the dissemination of the findings. The details on how we address these issues are provided below.

Process of recruitment and gaining informed consent. All participants will receive an information sheet explaining research activities taking place during the study, what will happen if they take part, the risk and benefits of their participation and confidentiality, and providing contact details of the research team. Participants will be given the time to reply to study invitation and/or discuss it with others if they wish to. All participants will be asked to consent that they have read and understood the information sheet. If participants wish to withdraw from the study, they will be free to do so at any time.

Practitioners. The practitioners will have up to 48 hours to decide whether they would like to participate after receiving the SIP. They will be also provided the research team contact details and will be asked to contact their research team, if they have any questions regarding their participation in the study. If the research team does not hear from them within 48 hours of receiving the SIP, a member of the research team will contact the practice manager to ask for the practitioner's decision to study participation.

Patients. Patients during proactive recruitment they will be given two weeks after invitation, before they are approached by a member of the practice staff to request their decision to participate. Patients approached opportunistically and interested to take part, they will be provided with the option to either proceed with the consent process or to take the time and think about their participation in the study. If patients prefer to take the time to think about their participation, they will be provided with up to 48 hours to decide whether they would like to participate. After that time the practice staff will contact them again to ask their decision to participate.
If patients have more questions about the research procedures, and these have not been answered by the recruitment material or the practice staff (e.g., practice staff during follow up calls to postage or practitioners during the face-to-face recruitment), they will be asked their verbal consent to pass their contact details to the research team. The research team will contact these patients, respond to any questions they may have, obtain their decision to participate, and, if interested, arrange how to obtain informed consent.

All participants. If any discomfort, distress, intrusion or inconvenience is expressed immediately before or during the recruitment and informed consent process, they will be asked if they would like to take some time before continuation, to reschedule the meeting or cease participation. If they would like to leave and not reschedule, the meeting will be stopped or cancelled. The same procedure will

be applied for the follow up interviews. All participants will be informed that they are free to withdraw from the study without giving a reason and without any adverse consequences.

If a participant expresses dissatisfaction with the study and wishes to seek advice, they will be given the contact details of the research team and the Patient Experience Team, NHS Cambridgeshire and Peterborough CCG, Lockton House, Clarendon Road, Cambridge, CB2 8FH, Tel: 0800 279 2535, Email: CAPCCG.pet@nhs.net

The research team will collect data on potential adverse events (AEs) that could occur and which may include, for example:

- breach of confidentiality
- complaints about any aspect of treatment as a study participant
- deviation from study protocol.
- aggressive behaviour of a participant towards the researcher, staff or others.

Data protection and participants' confidentiality.

Participant identifiable data expressing interest to participate in the study will be used only for the purpose of contacting the study participants. Express of interest (for patients) and electronic consent forms (for practitioners) will be obtained through Qualtrics, which is operated by the University secure network. The research team will only hold the information of participants providing informed consent. Written copy of participants' consent forms will be held by the research team in a secure filing cabinet in the research centre.

Participant identifiable data including participant names, emails and telephone numbers will be stored on a secure network to allow the research team to contact participants during the study. This information will be kept in a secure data hosting server in the research study centre, accessible only by research staff by two-way authentication, and held in compliance with the School of Clinical Medicine's information governance policy for sensitive and personal data (see: https://www.medschl.cam.ac.uk/research/information-governance/information-governance-policy/).

To set up the text message and app, patients' personal identifiable information will be obtained. This include their name, mobile phone number, prescribed medications, and CMG. This information will be transferred to Clinical School Servers (mySQL) using encrypted files through the internet.

In case of the app, additional information will be collected using app sensors. This includes accelerometer data and wifi usage: the accelerometer sensor collects data for user movement; the wifi sensor collects data about the near wifi, when and how often the participants' logs in. This data, and data about patients' usage of the app, will be stored in the app at patients' mobile phone only; and the app will not have functionalities that link patients' data to any other app or other social media service (e.g., facebook). Patients' information will be transferred to mySQL server, using encrypted internet files, for the purposes of analysis, and will be deleted from users' phone after that point.

The log file that contains patients' text message/app notification interaction and usage will be examined by the research team, who will remove telephone numbers or any other identifiable data, prior to analysis. After the study ends, all personal identifiable data will be anonymised using unique identification numbers, which will only be accessible by the research team.

Face to face consultation and follow up interviews will be audiotaped. Audios will be transcribed verbatim, using a professional external transcription service. This transcription service is well known to the research centre having been used in previous research studies. To ensure the safety of data, all data will be transferred through a secure encrypted web page. Audio recordings will be kept on the School of Clinical Medicine's secure server, separate from participants' personal data file. Transcripts will be examined by the research team and any identifiable personal information within the transcripts will be removed. Each participant will be given a reference number to be used in the transcripts. When all interviews are analysed, we will retain only the transcripts, all personal details and digital recording will not be retained. Normal practice of confidentiality, security and retention of research data will be observed.

Using the similar process reported above, data obtain from log files (e.g., text or app usage) will be saved in a secure and encrypted Cambridge University servers.
Participants' contact details will be retained during the process of data collection. Participants' identifiable personal data (in written format), will be kept in a locked cabinet, separate from transcripts and log files within the Institute of Public Health building at the University of Cambridge, which is ID card secured through double doors.

Telephone contacts with participants will be made through the University of Cambridge telephone system. Electronic communication (e.g., emails) will be conducted through a secure firewall protected network, provided by the University of Cambridge. Electronic communication (e.g., emails) with the

practices that involve the transfer of participants' personal data, will be conducted through an NHS secure firewall protected network.

All information collected during the course of the research will be kept strictly confidential and processed in accordance with the General Data Protection Regulation (GDPR) 2018 and according to the research unit and the University of Cambridge policy on data security policy. All participants will be given written information on the procedure being followed (see attached information sheet).

Research Ethics Committee (REC) and other Regulatory review & reports

Before the start of the study, a favourable opinion will be sought from a REC for the study protocol, informed consent forms and other relevant documents.

Before any site can enrol patients into the study, the Chief Investigator/Principal Investigator or designee will ensure that appropriate approvals from participating organisations are in place.

Peer review: this research protocol has been reviewed by the research team and the NIHR. Comments have been addressed and integrated in the final versions of the documents.

PATIENT AND PUBLIC INVOLVEMENT

Study materials including participant information sheets and consent forms have been reviewed by members of the PPI Addenbrooke's Hospital. Comments have been addressed and integrated in the final versions of the documents.

Competing Interests for the Chief Investigator: none

Insurance/Indemnity: The University of Cambridge insurance office has been consulted to ensure appropriate insurance/indemnity arrangements are in place to meet the potential legal liability of the Sponsor, investigators/collaborators arising from harm to participants in the design, management and conduct of the research.

Post-trial care: N/A

Access to the Outcome Data: the chief investigator and co-investigators will have access to the full dataset.

DISSEMINATION STRATEGY

The results of this research project will be written up for publication in peer-reviewed journals. Findings will also be disseminated through national and international conference presentations and other scientific meetings. If they request, patients will be sent a summary of the study findings. A report with results and recommendations will be sent to all practices taking part in the study. Sutton and Kassavou will lead on writing up for publication and dissemination.

REFERENCES

1. Public Health England (2018). Tackling high blood pressure: an update. (accessed online Nov 2018) https://assets.publishing.service.gov.uk/government/uploads/system/uploads/attachment_data/file/672554/Tackling_high_blood_pressure_an_update.pdf
2. World Health Organization. Adherence to long-term therapies: Evidence for action. WHO, Switzerland. 2003.
3. York Health Economics Consortium & School of Pharmacy, University of London. Evaluation of the scale, causes and costs of waste medicines. Report commissioned by the Department of Health, 2010.
4. Mazzaglia G, Ambrosioni E, Alacqua M, Filippi A, Sessa E, Immordino V, Borghi O, Caputi AP, Cricelli C, Mantovani LG. Adherence to antihypertensive medications and cardiovascular morbidity among newly diagnosed hypertensive patients. Circulation 2009;120:1598-1605.
5. Nieuwlaat R, Wilczynski N, Navarro T, Hobson N, Jeffery R, Keepanasseril A, Agoritsas T, Mistry N, Iorio A, Jack S, Sivaramalingam B, Iserman E,Mustafa RA, Jedraszewski D, Cotoi C, Haynes RB. Interventions for enhancing medication adherence. Cochrane Database of Systematic Reviews 2014, Issue 11. Art. No.: CD000011. DOI: 10.1002/14651858.CD000011.pub4.
6. National Institute for Health and Care Excellence. Medicines adherence: Involving patients in decisions about prescribed medicines and supporting adherence. NICE clinical guideline 76. NICE, London, 2009.
7. NICE (2011). Hypertension in adults: diagnosis and management. Accessed online Nov 2018 https://www.nice.org.uk/guidance/cg127
8. NICE (2016). Physical activity: brief advice for adults in primary care. (accessed online Nov 2018) https://www.nice.org.uk/guidance/ph44
9. NICE (2018). Stop smoking interventions and services. (accessed online Nov 2018) https://www.nice.org.uk/guidance/ng92

10. Ofcom. (2012). The text message is 20 years old today. Retrieved from https://www.ofcom.org.uk/about-ofcom/latest/media/media-releases/2012/the-text-message-is-20-years-old-today
11. Poushter, J. (2016). Smartphone ownership and internet usage continues to climb in emerging economies. Pew Research Center, 22, 1-44.
12. Glynn LG, Murphy AW, Smith SM, Schroeder K, Fahey T. Interventions to improve control of blood pressure in patients with hypertension. Cochrane Database of Systematic Reviews 2010, Issue 3. Art. No.: CD005182. DOI:10.1002/14651858.CD005182.pub4.
13. Clark, Smith, Taylor, Campbell. Nurse led interventions to improve control of blood pressure in people with hypertension: systematic review and meta-analysis. BMJ. 2010 Aug 23;341:c3995. doi: 10.1136/bmj.c3995.
14. Laurant M, van der Biezen M, Wijers N, Watananirun K, Kontopantelis E, van Vught A JAH. Nurses as sub-stitutes for doctors in primary care. Cochrane Database of Systematic Reviews 2018, Issue 7. Art. No.: CD001271. DOI:10.1002/14651858.CD001271.pub3.
15. Schroeder K, Fahey T, Ebrahim S. Interventions for improving adherence to treatment in patients with high blood pressure in ambulatory settings. Cochrane Database of Systematic Reviews 2004, Issue 3. Art. No.: CD004804. DOI:10.1002/14651858.CD004804.
16. Farmer A, Hardeman W, Hughes D, Prevost AT, Kim Y, Craven A, Oke J, Boase S, Selwood M, Kellar I, Graffy J, Griffin S, Sutton S, Kinmonth AL. An explanatory randomised controlled trial of a nurse-led consultation-based intervention to support patients with adherence to taking glucose lowering medication for type 2 diabetes. BMC Family Practice 2012 13:30.
17. Thakkar, J., Kurup, R., Laba, T. L., Santo, K., Thiagalingam, A., Rodgers, A., ... & Chow, C. K. (2016). Mobile telephone text messaging for medication adherence in chronic disease: a meta-analysis. JAMA internal medicine, 176(3), 340-349.
18. Adler, A. J., Martin, N., Mariani, J., Tajer, C. D., Owolabi, O. O., Free, C., ... & Perel, P. (2017). Mobile phone text messaging to improve medication adherence in secondary prevention of cardiovascular disease. Cochrane database of systematic reviews, (4).
19. Kassavou, A., & Sutton S. (2017) Automated telecommunication interventions to promote adherence to cardio-metabolic medications: meta-analysis of effectiveness and meta-regression of behaviour change techniques. Health Psychol Rev. 12:1-18.
20. Bobrow, K., Farmer, A. J., Springer, D., Shanyinde, M., Yu, L. M., Brennan, T., ... Levitt, N. (2016). Mobile phone text messages to support treatment adherence in adults with high blood pressure (SMS-Text Adherence Support [StAR]): A single-blind, randomized trial. Circulation, 133(6), 592–600. doi:10.1161/CIRCULATIONAHA.115.017530

21. Kassavou, A., Mirzaei, V., Brimicombe, J, Edwards, S., Massou, E., Prevost, T., Griffin, S. & Sutton S. A highly tailored text and voice messaging intervention to improve medication adherence in patients with either or both hypertension and type 2 diabetes in the UK primary care setting. A feasibility randomised controlled trial of clinical effectiveness. J Med Internet Res 2020;22(5):e16629 doi: 10.2196/16629
22. Armitage, L., Kassavou, A., & Sutton, S. (2018). The use of mobile applications to support medication adherence. A systematic review of the literature with meta-analysis. manuscript in preparation.
23. Barber, N. Should we consider non-compliance a medical error? Qual Saf Health Care, 2002, 11(1), 81-84.
24. Sutton, S. (2015). Stage theories. In M. Norman (Ed.), Predicting and changing health behaviour: Research and practice with social cognition models. Buckingham: Open University Press.
25. Kassavou A, & Sutton S. (2016). Developing and pre-testing a tailored interactive voice response (IVR) intervention to support adherence to anti-hypertensive medications: a research protocol. Cambridge.
26. Kassavou, A., Houghton, V., Edwards, S., Wilson E., Brimicombe, J., Griffin, S., Sutton, S. Acceptability of the Medication Adherence for Patients Support (MAPS) intervention to improve adherence to patients prescribed multiple medications, as an adjunct to primary care: a qualitative study. Journal of Health Psychology
27. Kassavou, A., Houghton, V., Edwards, S., Brimicombe, J., Sutton, S. (2019) Development and piloting of a highly tailored text and voice messaging intervention to support adherence to antihypertensive medications within the primary care setting. accepted for publication in BMJ Open
28. Tomaszewski, M, White, C., Patel, P., Masca, N., Damani, R, Hepworth, J., Samani, NJ., Gupta, P., Madira, W., Stanley, A., Williams, B. (2014). High rates of non-adherence to antihypertensive treatment revealed by HP LC-MS/MS urine analysis. Heart 100(11):855-61. doi: 10.1136/heartjnl-2013-305063
29. Mora P, Berkowitz A, Contrada RJ, Wisnivesky J, Horne R, Leventhal H, Halm EA. Factor structure and longitudinal invariance of the Medication Adherence Report Scale-Asthma. Psychology and Health 2011;26:713-27.
30. The EuroQol Group: EuroQol- a new facility for the measurement of health-related quality of life. *Health Policy* 1990, 16:199-208.
31. Horne R, Weinman J, Hankins M: The Beliefs about Medicines Questionnaire: the development and evaluation of a new method for assessing the cognitive representation of medication. Psychol Health 1999, 14:1-24.

32. McManus RJ, Mant J, Bray EP, Holder R, Jones MI, Greenfield S, Kaambwa B, Banting M, Bryan S, Little P, Williams B, Hobbs FDR. Telemonitoring and self-management in the control of hypertension (TASMINH2): a randomised controlled trial. Lancet 2010;376:163-72.
33. MRC Epidemiology Unit. Measuring blood pressure with Omron 7051T. Unpublished document.
34. Kassavou, A., Turner, A., French D.P. (2015). The role of walkers' needs and expectations in supporting maintenance of attendance at walking groups: a longitudinal multi-perspective study of walkers and walk group leaders. PLoS One. 16;10(3):e0118754.
35. Braun, V., & Clarke, V. (2012). Thematic Analysis. APA Handbook of Research Methods in Psychology: Volume 2, 2, 57–71. http://doi.org/10.1037/13620-004
