# Supplementary material for: The feasibility of the PAM intervention to support treatment-adherence in people with hypertension in primary care: a randomised clinical controlled trial
Source: Sci Rep. 2021 Apr 26;11:8897. doi: 10.1038/s41598-021-88170-2 (PMC8076273; doi:10.1038/s41598-021-88170-2)
Supplement: Supplementary file 2 [file 41598_2021_88170_MOESM2_ESM.docx]

**Supplementary file 2**

**The feasibility of PAM intervention to support treatment-adherence in people with hypertension in primary care. A randomised clinical controlled trial.**

Aikaterini Kassavou^1*^, Venus Mirzaei^1^, Sonia Shpendi^1^, James Brimicombe^1^, Jagmohan Chauhan^2,3^, Debi Bhattacharya^4^, Felix Naughton^5^, Wendy Hardeman^5^, Helen Eborall^6^, Miranda Van Emmenis^1^, Anna De Simoni^7^, Amrit Takhar^8^, Pankaj Gupta^9^, Prashanth Patel^9^, Cecilia Mascolo^2^, Andrew Toby Prevost^10^, Stephen Morris^1^, Simon Griffin^1^, Richard J McManus^11^, Jonathan Mant^1^ & Stephen Sutton^1^

^1^Department of Public Health and Primary Care, the Primary Care Unit, University of Cambridge

^2^Department of Computer Science and Technology, University of Cambridge

^3^School of Electronics and Computer Science, University of Southampton

^4^School of Pharmacy, University of East Anglia

^5^School of Health Sciences, University of East Anglia

^6^Usher Institute, University of Edinburgh

^7^ Institute of Population Health Sciences, Queen Mary University of London

^8^Cambridgeshire and Peterborough Clinical Commissioning Group

^9^Department of Metabolic Medicine and Chemical Pathology, University Hospitals of Leicester NHS Trust

^10^Nightingale-Saunders Clinical Trials and Epidemiology Unit, King’s College London

^11^Nuffield Department of Primary Care Health Sciences, University of Oxford

*corresponding author email [kk532@medschl.cam.ac.uk](mailto:kk532@medschl.cam.ac.uk)

**Table 1.** Unadjusted outcome data at 3-month follow up.

| **Variables** | | **Intervention** | **Control** |
| --- | --- | --- | --- |
| Blood pressure, mean (SD), mm Hg | |  |  |
|  | Systolic | 136.9 (10.30) | 145.9 (5.19) |
|  | Diastolic | 79.55 (10.69) | 84.6 (8.47) |
| Full lipid profile, mean (SD), mmol/mol | |  |  |
|  | Cholesterol | 4.23 (0.75) | 5.55 (1.59) |
|  | Triglycerides | 1.62 (1.08) | 2.00 (0.47) |
|  | HDL Cholesterol | 1.69 (0.59) | 1.90 (0.48) |
|  | LDL Cholesterol | 1.79 (0.38) | 2.28 (0.70) |
| Glycated haemoglobin, mean (SD), mmol/mol | | 46.00 (11.22) | 48.78 (10.59) |
| Medication Adherence | |  |  |
|  | Biochemically validated, percent (frequency) | 96 (48) | 91.2 (31) |
|  | Self-reported, adherence past week, means (SD) | 6.96 (0.19) | 6.06 (0.91) |
|  | Self-reported, adherence past month, means (SD) | 9.84 (0.36) | 8.56 (1.48) |
| Quality of Life, median (min-max), EQ-5D-5L | |  |  |
|  | Mobility | 1 (1-1) | 2 (1-3) |
|  | Self-care | 1 (1-1) | 1 (1-5) |
|  | Usual activity | 1 (1-4) | 1 (1-5) |
|  | Pain/ discomfort | 1 (1-3) | 2 (1-5) |
|  | Anxiety/ depression | 1 (1-2) | 1 (1-4) |
| Total health, mean (SD), EQ-5D-5L | | 85.18 (16.82) | 72.69 (23.64) |
| Beliefs about Medicines, mean (SD) | |  |  |
|  | Generic beliefs | 23.78 (3.58) | 23.15 (4.35) |
|  | Concern beliefs | 17.48 (3.46) | 17.07 (3.85) |
|  | Necessity beliefs | 13.57 (3.77) | 10.69 (3.43) |
| Number of defined daily doses of medication prescribed for hypertension, median (min – max) | | 2.00 (1-6) | 3.00 (1-6) |

Complete case analysis. N=84 (n=50 intervention, n=34 control); blood pressure, urine samples, self-reported medication adherence. N=68 (n=42 intervention and n=26 control) EQ-5D-5L and Beliefs about Medicines Questionnaire. N=42 full lipid profile (n=28 intervention, n=14 control). N=34 Glycated haemoglobin (n=23 intervention, n=11 control).

**Table 2**. Mean change in Blood Pressure treatment by practice level variability

| **Variables** | | **beta** | **95% CI** | |
| --- | --- | --- | --- | --- |
| Practice practitioner, nurse | |  | Lower | Upper |
|  | 1 | -1.40 | -15.30 | 12.50 |
|  | 2 | 1.60 | -8.67 | 11.87 |
|  | 3 | 3.10 | -6.27 | 12.47 |
|  | 4 | 1.86 | -3.76 | 7.48 |
|  | 5 | -0.75 | -8.99 | 7.47 |
|  | 6 | 2.88 | -5.34 | 11.12 |
|  | 7 | -1.86 | -8.39 | 4.67 |
|  | 8 | -1.20 | -16.30 | 11.50 |
|  | 9 | -2.40 | -15.49 | 10.03 |
| Practice IMD | |  |  |  |
|  | 10-30 | 2.78 | -3.55 | 9.13 |
|  | 40-60 | 3.72 | -2.35 | 9.80 |
|  | 70-100 | 3.58 | -3.07 | 10.25 |

Dependent variable: Mean changes in Systolic Blood Pressure

Independent Variables: Practitioner, Index of Multiple Deprivation


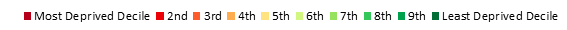


| **Practice ID** | 1 | 2 | 3 | 4 | 5 | 6 | 7 | 8 | 9 |
| --- | --- | --- | --- | --- | --- | --- | --- | --- | --- |
| **Number of invited eligible patients** | 752 | 282 | 38 | 457 | 389 | 394 | 796 | 400 | 351 |
|  | | | | | | | | | |

**Figure 1**. Index of multiple deprivation and number of invited patients per primary care practice. Total number of eligible patients invited in this trial N=3,859. Practice 2,3,6,7,8 were in East of England. Practice 1,4,5,9 were in London.

**
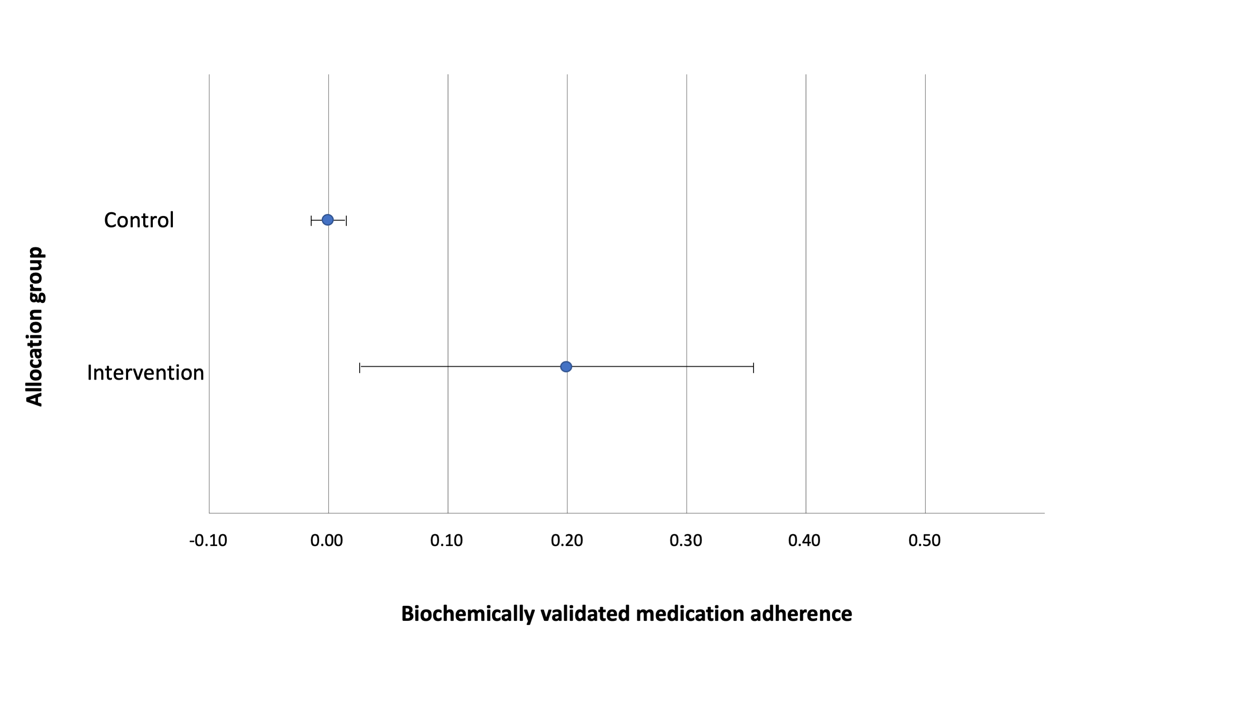
**

**Figure 2**. Improvements in biochemically validated medication adherence per group

The horizontal axis shows the percentage of improvement in daily prescribed doses. Higher numbers show improvements in biochemically validated medication adherence. Vertical axis shows allocation group. The dots show the mean change, and the lines show the 95% Confidence Intervals.


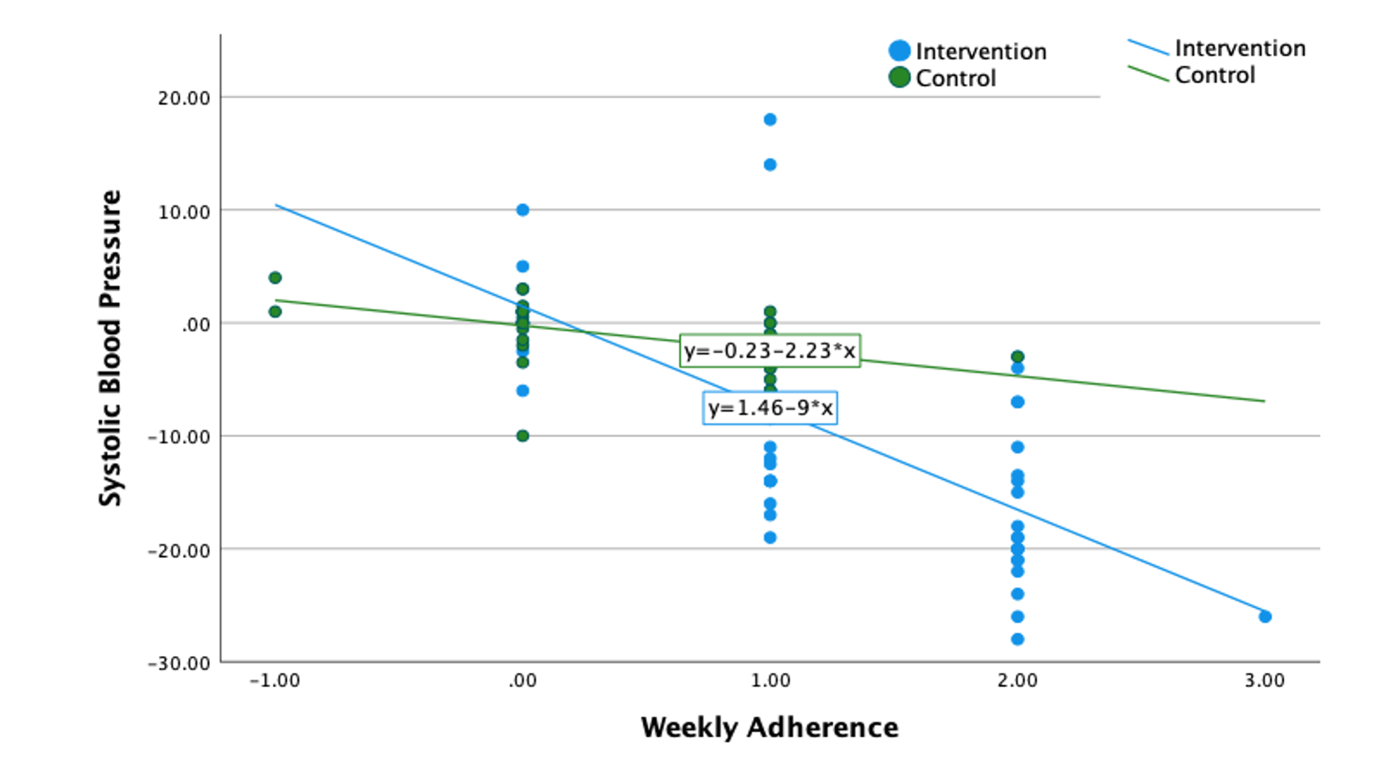


**Figure 3.** Improvements in systolic blood pressure, per days of medication adherence and group

The horizontal axis shows the changes in days of medication adherence per week. Higher numbers show more days of adherence during the past week. Vertical axis shows the changes in Systolic Blood Pressure. Lower numbers indicate reduction in Systolic Blood Pressure. The green line is the fitted regression line for the control group, the blue line is the fitted regression line for the intervention group. The dots show the units of measure for each of the intervention or the control group. The direction of the lines shows the association between changes in days of adherence and changes in systolic blood pressure.

**
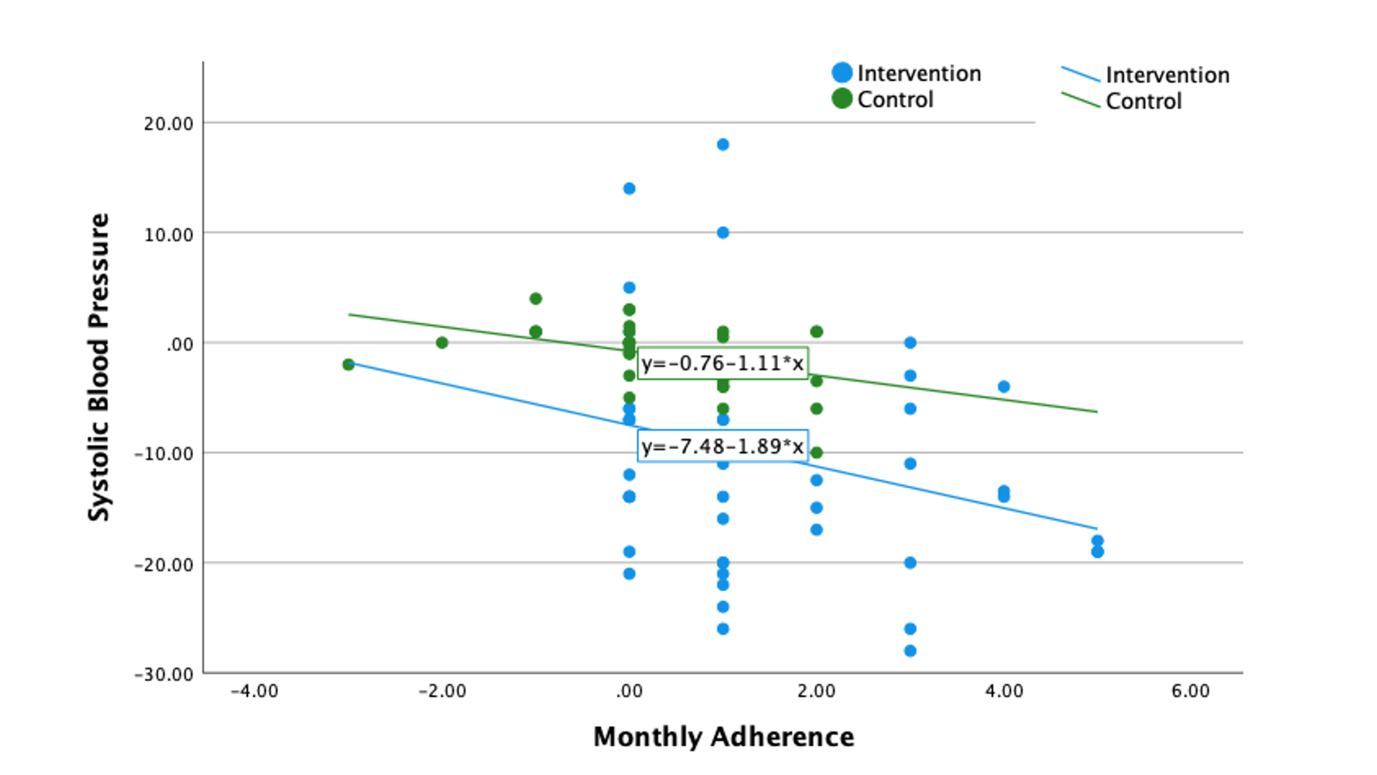
**

**Figure 4.** Improvements in systolic blood pressure, per monthly medication adherence and group

The horizontal axis shows the changes in percent of adherence per month. Higher numbers show improvement in adherence during the past month. The vertical axis shows the changes in Systolic Blood Pressure. Lower numbers show reduction in Systolic Blood Pressure. The green line is the fitted regression line for the control group, the blue line is the fitted regression line for the intervention group. The dots show the units of measure for each of the intervention and the control group. The direction of the lines shows the association between changes in medication adherence per month and changes in systolic blood pressure.


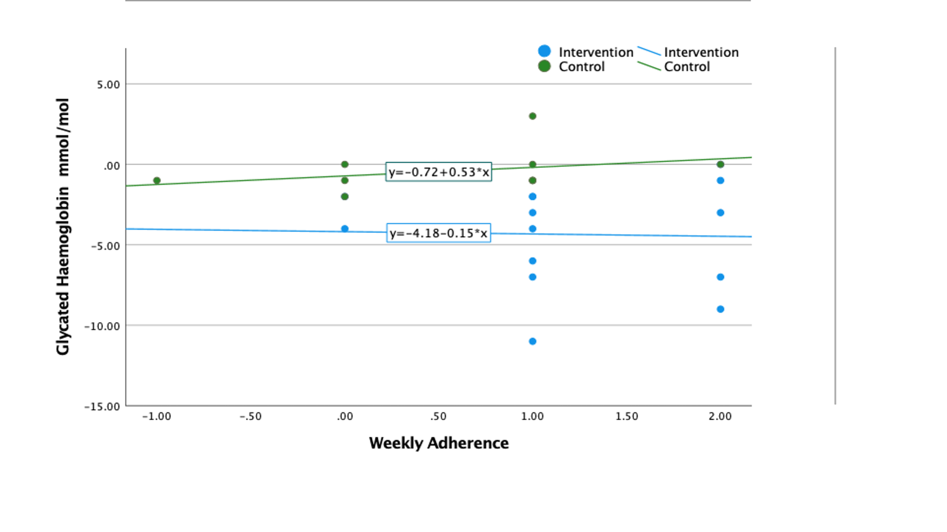

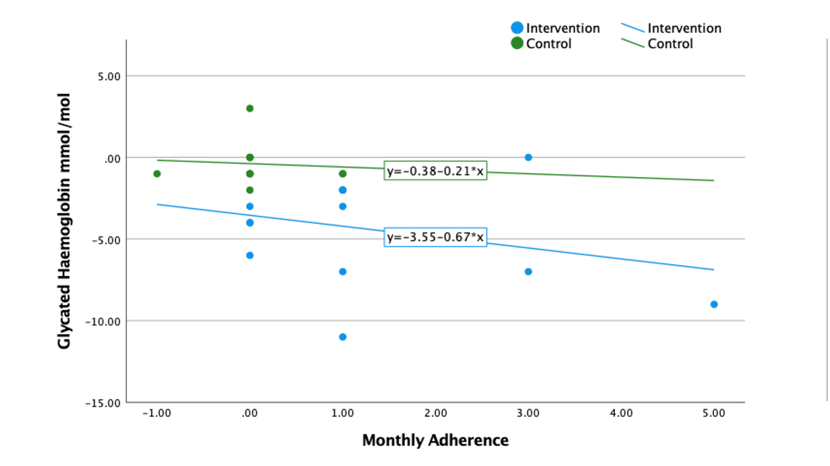


b

a

**Figure 5**. Improvements in glycated haemoglobin, per medication adherence and group

The vertical axis shows the changes in glycated haemoglobin. Lower numbers show reduction in glycated haemoglobin. The horizontal axis shows the changes in (a) days of medication adherence, or (b) percent of adherence per month. Higher numbers show improvement in medication adherence. The green line is the fitted regression line for the control group, the blue line is the fitted regression line for the intervention group. The dots show the units of measure for each of the intervention or the control group. The direction of the lines shows the association between changes in adherence and changes in glycated haemoglobin.


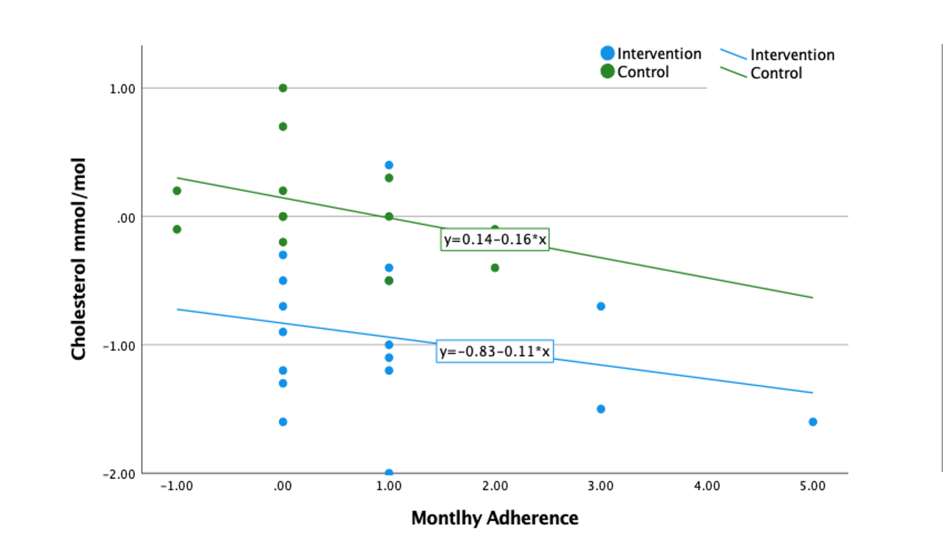

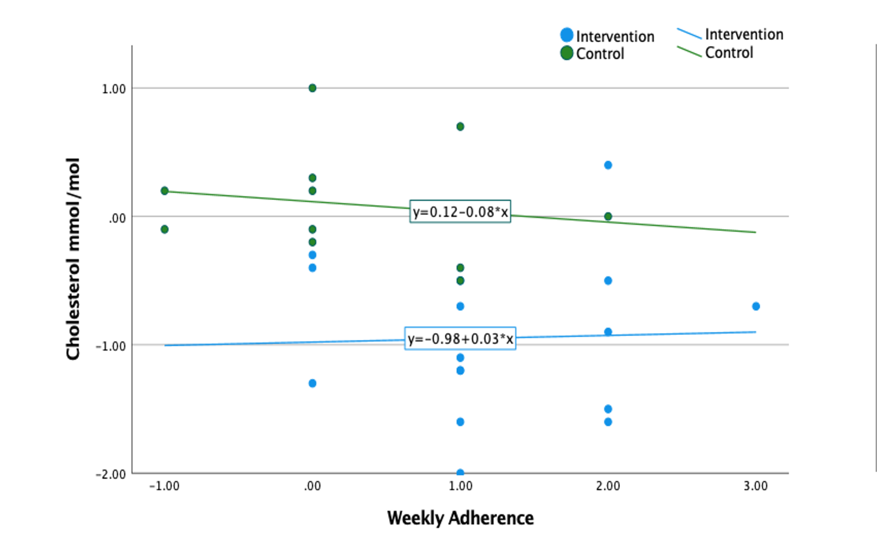


b

a

**Figure 6**. Improvements in cholesterol, per medication adherence and group

The vertical axis shows the changes in cholesterol. Lower numbers show reduction in cholesterol. The horizontal axis shows the changes in (a) days of medication adherence, or (b) percent of adherence per month. Higher numbers show improvement on medication adherence. The green line is the fitted regression line for the control group, the blue line is the fitted regression line for the intervention group. The dots show the units of measure for each of the intervention or the control group. The direction of the lines shows the association between changes in adherence and changes in cholesterol.
